# Temporal dissociation of COX-2-dependent arachidonic acid and 2-arachidonoylglycerol metabolism in RAW264.7 macrophages

**DOI:** 10.1016/j.jlr.2024.100615

**Published:** 2024-08-05

**Authors:** Ansari M. Aleem, Michelle M. Mitchener, Philip J. Kingsley, Carol A. Rouzer, Lawrence J. Marnett

**Affiliations:** A. B. Hancock, Jr., Memorial Laboratory for Cancer Research, Departments of Biochemistry, Chemistry and Pharmacology, Vanderbilt Institute of Chemical Biology, and Vanderbilt-Ingram Cancer Center, Vanderbilt University School of Medicine, Nashville, TN, USA

**Keywords:** Kdo2-lipid A, TLR4, lipidomics, prostaglandins, prostaglandin glyceryl esters, arachidonic acid, 2-arachidonoylglycerol, DAG lipase, macrophages

## Abstract

Cyclooxygenase-2 converts arachidonic acid to prostaglandins (PGs) and the endocannabinoid, 2-arachidonoylglycerol (2-AG), to PG glyceryl esters (PG-Gs). The physiological function of PG biosynthesis has been extensively studied, but the importance of the more recently discovered PG-G synthetic pathway remains incompletely defined. This disparity is due in part to a lack of knowledge of the physiological conditions under which PG-G biosynthesis occurs. We have discovered that RAW264.7 macrophages stimulated with Kdo2-lipid A (KLA) produce primarily PGs within the first 12 h followed by robust PG-G synthesis between 12 h and 24 h. We suggest that the amount of PG-Gs quantified is less than actually synthesized, because PG-Gs are subject to a significant level of hydrolysis during the time course of synthesis. Inhibition of cytosolic phospholipase A2 by giripladib does not accelerate PG-G synthesis, suggesting the differential time course of PG and PG-G synthesis is not due to the competition between arachidonic acid and 2-AG. The late-phase PG-G formation is accompanied by an increase in the level of 2-AG and a concomitant decrease in 18:0-20:4 diacylglycerol (DAG). Inhibition of DAG lipases by KT-172 decreases the levels of 2-AG and PG-Gs, indicating that the DAG-lipase pathway is involved in delayed 2-AG metabolism/PG-G synthesis. These results demonstrate that physiologically significant levels of PG-Gs are produced by activated RAW264.7 macrophages well after the production of PGs plateaus.

## Graphical Abstract

**Figure:**
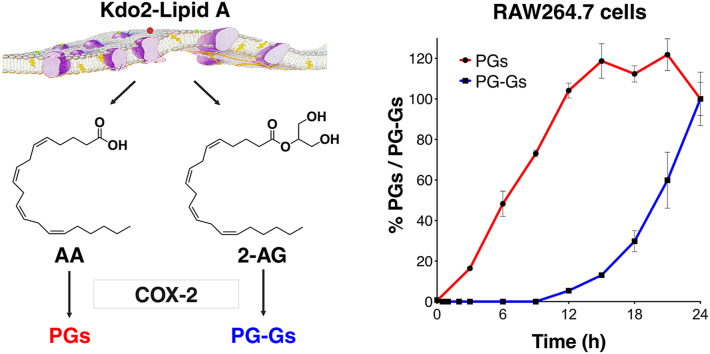

Arachidonic acid (AA) is a component or precursor of many bioactive lipids including lysophospholipids, diacylglycerol (DAG), endocannabinoids, prostaglandins (PGs), leukotrienes, lipoxins, and epoxyeicosatrienoic acids (1, 2). Hydrolases and acyltransferases account for the formation of lysophospholipids, DAG, and endocannabinoids whereas oxygenases catalyze key steps in the formation of PGs, leukotrienes, lipoxins, and epoxyeicosatrienoic acids. Oxygenation of AA by PG G/H synthases, colloquially known as cyclooxygenases (COX-1 and COX-2), produces PGG_2_, which is then reduced to PGH_2_ at a separate site on the COX protein (3). PGH_2_ is converted by downstream enzymes into PGE_2_, PGD_2_, PGF_2α_, PGI_2_, and thromboxane A_2_ (TXA_2_) (Fig. 1) (4, 5). PG and TxA_2_ actions are mediated by binding to one or more membrane-bound G protein-coupled receptors (6). In addition, PGD_2_ undergoes multiple nonenzymatic dehydrations to form a series of cyclopentenone PGs, including PGJ_2_, Δ^12^-PGJ_2_, and 15-deoxy-Δ^12,14^-PGJ_2_ (15d-PGJ_2_) (supplemental Fig. S1) (7, 8). The cyclopentenone PGs are reactive electrophiles and covalently modify cysteine residues in the ligand-binding domains of nuclear transcription factors such as peroxisome proliferator-activated receptor gamma, resulting in transcriptional activation (9). PGs are not stored but are synthesized on demand following activation of phospholipase A2 (PLA2) enzymes, which catalyze the release of AA from phospholipid stores (10). Consequently, the levels of PGs increase dramatically following cell stimulation due to activation of PLA2 and, in some cases, induction of COX enzyme expression (mainly COX-2) (11).

**Fig. 1:**
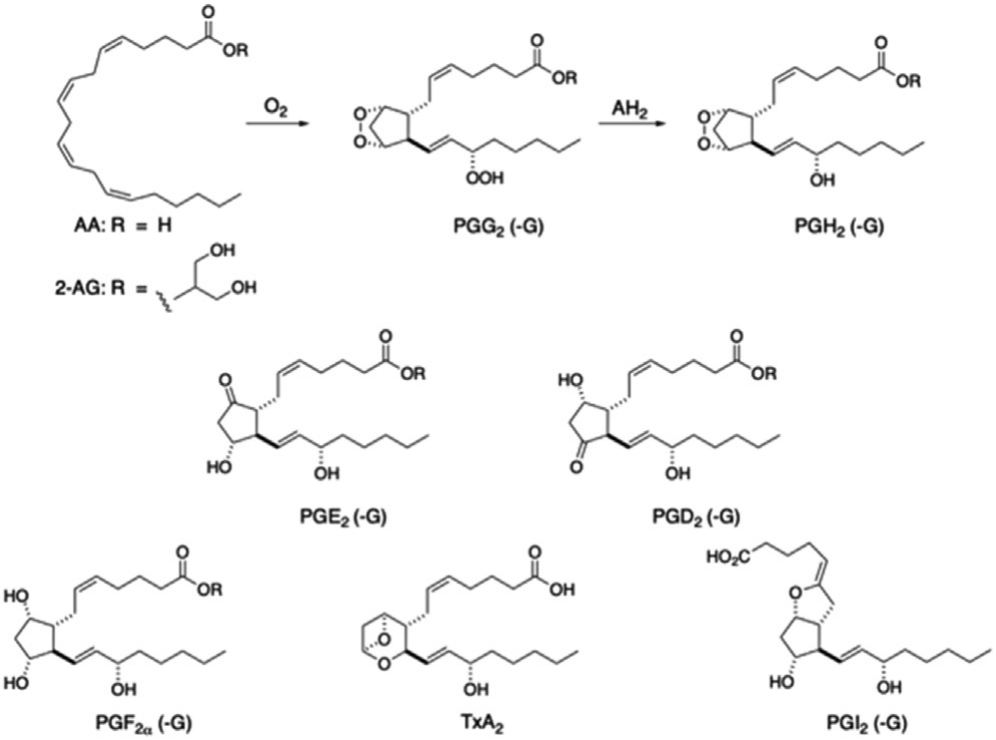
Production of prostanoids via the cyclooxygenase pathway. AA or 2-AG is oxygenated to PGG_2_ or PGG_2_-G, respectively, which are then reduced to PGH_2_ or PGH_2_-G using electrons from a generic reductant, AH_2_. PGH_2_ is converted to PGs, and PGH_2_-G is converted to PG-Gs. TXA_2_ is formed from PGH_2_. 2-AG, 2-arachidonoylglycerol; AA, arachidonic acid; PG, prostaglandin; PG-Gs, PG glyceryl esters; TXA_2,_ thromboxane A_2_.

COX-2 oxygenates a broader range of substrates than COX-1 (12, 13). Foremost among them is the endocannabinoid 2-arachidonoylglycerol (2-AG, Fig. 1), which is primarily synthesized from AA-containing DAGs through the action of DAG lipases (DAGLα and β) (14). COX-1 is capable of oxygenating 2-AG, but COX-2 is ∼20-fold more efficient (13). The product of 2-AG oxygenation is the glyceryl ester of PGH_2_ (PGH_2_-G), which is a substrate for four of the five endoperoxide-metabolizing enzymes; it is a poor substrate for TxA synthase. Thus, 2-AG oxygenation by COX enzymes results in the formation of PGE_2_-G, PGD_2_-G, PGF_2α_-G, and PGI_2_-G (15). These compounds exert potent biological activities, some of which are mediated by PG receptors but others through non-PG receptors (16, 17, 18, 19, 20, 21). For example, PGE_2_-G binds to and activates the purinergic receptor P2Y_6_ at concentrations five orders of magnitude lower than its cognate ligand, UDP (22). It is important to note that 2-AG itself is a ligand for the cannabinoid receptors, CB1 and CB2, so its oxygenation by COX-2 can both alter tone at the CB receptors *AND* generate PG glyceryl esters (PG-Gs), which have their own biological activity (23).

The role of bioactive lipids in the activation and resolution of inflammation is a topic of intense current interest. Macrophages play an important role in both processes and are prodigious producers of PGs following stimulation with a range of agonists such as lipopolysaccharide (LPS) or zymosan. The biosynthesis of PGs and their involvement in the inflammatory response has been extensively studied, and its importance is underscored by the fact that non-steroidal anti-inflammatory drugs exert their pharmacological actions primarily by binding to and inhibiting COX enzymes (24, 25, 26, 27, 28, 29). In contrast, detailed analyses of the biosynthetic pathways associated with PG-Gs in macrophages are lacking. A few studies have demonstrated the ability of primary and cultured macrophages to synthesize PG-Gs from 2-AG following stimulation with LPS and zymosan. These include resident peritoneal macrophages (RPMs) and macrophage cell lines such as J774 and RAW264.7 (19, 30, 31).

We chose to explore PG-G synthesis in activated RAW264.7 cells. This cell line was isolated from a murine tumor generated by Abelson leukemia virus in an animal treated with pristane (32). It has been extensively used for investigations of lipid metabolism in the context of inflammation including by two large research consortia (the Alliance for Cellular Signaling and Lipid Maps). The Lipid Maps consortium generated a massive body of publicly available data on the metabolic consequences of stimulating RAW264.7 cells with LPS or its biologically active precursor, Kdo2-lipid A (KLA) (https://www.lipidmaps.org). However, no analysis of 2-AG or PG-G metabolism was reported in any of the deposited datasets. In the current study, we provide a detailed description of lipid mediator synthesis through the COX pathway in RAW264.7 cells stimulated by KLA. We uncovered a significant difference in the time courses and amounts of PG and PG-G produced, and we explored various explanations for this temporal difference. The most likely pathway for PG-G production involves DAG hydrolysis to 2-AG which is then oxygenated by COX-2. These findings establish a foundation for investigation of endocannabinoid-COX crosstalk in macrophages activated during the inflammatory response.

## Materials and Methods

### Materials

AA (CAS 506-32-1), PGE_2_-d_4_ (CAS 34210-10-1), PGF_2α_-d_4_ (CAS 34210-11-2), PGJ_2_-d_4_ (CAS 2738376-80-0), 15dPGJ_2_-d_4_ (CAS 1542166-82-4), 15-deoxy-Δ^12,14^-PGD_2_-d_4_ (15dPGD_2_-d_4_, CAS 2750534-91-7), PGE_2_-1-glyceryl ester-d_5_ (PGE_2_-G-d_5_, Item No.10004197), AA-d_8_ (CAS 69254-37-1), 2-AG-d_5_ (CAS 2522598-88-3) and 1-stearoyl-2-arachidonyl-d_8_-*sn*-glycerol (Item No.10009872) were from Cayman Chemical. Authentic standards of analytes mentioned in supplemental Table S1 were also purchased from Cayman Chemical. PGD_2_-G-d_5_ was synthesized as described previously (13). IGEPAL® CA-630 was from MP Biomedicals. Mouse anti-COX-2 polyclonal antibody was from Cayman Chemical. Mouse anti-DAGLβ was from Cell Signaling Technology. Goat anti-actin (1–19) and mouse anti-cytosolic phospholipase A2 (cPLA2) antibodies were from Santa Cruz Biotechnology. IRDye® 800CW donkey anti-rabbit and IRDye® 680 donkey anti-goat antibodies were from LI-COR. KLA (3-deoxy-d-manno-octulosonic acid-lipid A) (CAS 1246298-62-3) was purchased from Avanti Polar Lipids. LC-grade solvents were purchased from Sigma-Aldrich. Giripladib was a kind gift from Alex Brown and Craig Lindsley. KT-172, ML-299, VU0155069, and ML-298 were purchased from Cayman Chemical. U73122 and U73343 were purchased for Tocris, and propranolol hydrochloride was from Sigma-Aldrich.

### Cell culture, stimulation, and treatment

RAW264.7 cells from the American Type Culture Collection (Rockville, MD) were maintained in Dulbecco’s Modified Eagle Medium with GlutaMAX™ containing 4.5 g/L D-glucose and 110 mg/L sodium pyruvate (Gibco, Grand Island, NY) supplemented with 10% heat-inactivated FBS (Atlas Biologicals, Fort Collins, CO) at 37°C and 5% CO_2_. Low passage number cells were counted on a TC-20 automated cell counter (Bio-Rad), plated onto 60-mm culture dishes, and allowed to adhere overnight (1 × 10^6^ cells in 3 ml medium). The cells were stimulated by replacing the medium with fresh medium/10% FBS containing 100 ng/ml KLA. The KLA was sonicated for 5 min in a water bath sonicator before addition to the medium. Inhibitors dissolved in dimethyl sulfoxide were added to the culture dish along with KLA (giripladib) or 12 h after activation (KT-172). An equal concentration of dimethyl sulfoxide served as a control.

### Sample preparation

At desired time points, the cells were scraped into the culture medium, and the suspension was subjected to centrifugation (500*g* × 5 min). The medium was removed, and two volumes of ethyl acetate (containing 1% glacial acetic acid and the appropriate deuterated internal standards) were added to extract eicosanoids. This mixture was vortexed and then centrifuged. The upper, organic layer was collected and evaporated to dryness under nitrogen gas. The samples were then reconstituted in 110 μl of 1:1 (v:v) methanol:water and analyzed by LC-MS/MS as described below. PGs analyzed included PGD_2_, PGE_2_, PGJ_2_, PGF_2α_, 15-deoxy-Δ^12-14^-PGD_2_ (15d-PGD_2_), and 15d-PGJ_2_. PGD_2_ and PGE_2_ are the major COX products of RAW264.7 cell stimulation; however, over time the dehydration/rearrangement products of PGD_2_ accumulate.

Cell pellets were used to determine the intracellular levels of AA, 2-AG, and selected DAG species, which were extracted into ice-cold methanol containing internal standards. After thorough vortexing, the samples were centrifuged at 3000 *g* for 20 min. The supernatant was collected and evaporated to dryness under nitrogen gas. The samples were then reconstituted in 150 μl of 2:1 (v:v) isopropanol:water and analyzed by LC-MS/MS. Note that AA is released extracellularly by activated RAW264.7 cells. For example, the LipidMaps consortium (https://www.lipidmaps.org) reported that intracellular and extracellular levels of AA were similar following KLA treatment, although the peak level occurred 30 min earlier and was 2-fold higher in the culture medium. In our studies, we focused on intracellular levels of these species because we were primarily interested in them as substrates for COX-2, an intracellular enzyme.

### Hydrolysis of PG-Gs

To determine the rate of PG-G hydrolysis, desired amounts of PGE_2_-G-d_5_ or PGD_2_-G-d_5_ were added to dishes containing medium only, medium plus cells, or medium plus cells plus KLA. An aliquot of medium was removed at the desired time points and added to two volumes of ethyl acetate containing 1% glacial acetic acid with 15d-PGJ_2_-d_4_ as internal standard. The samples were dried, resuspended, and analyzed via LC-MS/MS as described below.

### LC-MS/MS analysis

The LC-MS/MS systems used were either a Shimadzu LC unit connected in-line with a SCIEX 3200 QTrap mass spectrometer or a Shimadzu Nexera UPLC system in-line with a SCIEX 6500 QTrap mass spectrometer. SCIEX Analyst (version 1.6.2) was used for instrument control, data acquisition, and processing.

All analytes were chromatographed in reverse-phase mode with a gradient elution scheme. Furthermore, all analytes were detected via multiple reaction monitoring. The multiple reaction monitoring transitions for all analytes are given in supplemental Table S1. Representative LC-MS/MS chromatograms and relevant analytical details for all the analytes in this study are provided in supplemental Figs. S2–S5. Analytes were quantified via stable isotope dilution against their deuterated analogs unless otherwise indicated. Note that, as shown in supplemental Fig. S4, the values for 2-AG reported here actually represent a combination of 2-AG and a small quantity of 1(3)-AG, a product of nonenzymatic acyl migration.

### Western blot

For analysis of COX-2, cPLA2, and DAGLβ levels, cells harvested as described above were lysed by sonication in 150 mM NaCl, 50 mM Hepes, and 1% IGEPAL® CA-630. Following centrifugation at 21,000 rpm for 15 min at 4 °C, lysate proteins were resolved on a 4%–15% Mini-Protean TGX precast gel (Bio-rad). Proteins were transferred onto a nitrocellulose membrane and probed for the proteins using the aforementioned primary antibodies. Actin was used as a loading control. Proteins were visualized using IRDye® 800CW donkey anti-rabbit or IRDye® 680 donkey anti-goat using a Li-Cor Odessey Infrared Imaging System (https://www.licor.com/bio/odyssey-family).

### Quantitative RT-PCR

To quantify the effect of KLA on DAGLβ gene expression, KLA-stimulated cells were harvested at different time points following KLA addition, and RNA was extracted from the cells using TRIzol reagent. An iScript™ (Bio-Rad) cDNA synthesis kit was used to make the first strand of cDNA for analysis of gene expression, and iTaq Universal SYBR Green Supermix (Bio-Rad) was used for qPCR analysis as per the manufacturer’s instructions. The primers used for DAGLβ were (F: AGCGACGACTTGGTGTTCC; R: GCGTGAGATACAACGTCAGACT). GAPDH (F: TGCTGAGTATGTCGTGGAGT; R: GTTCACACCCATCACAAACA) was used as an internal reference gene to obtain relative expression quantification of DAGLβ.

### Statistical analysis

For data analysis and figures, GraphPad Prism (Version 10; GraphPad Software Inc., La Jolla, CA; https://graphpad.com) was used. One-way ANOVA was used for statistical analysis.

## Results

### Temporal difference in PG and PG-G synthesis in response to KLA

LPS stimulation of RAW264.7 cells through the toll-like receptor 4 initiates a signal transduction cascade leading to many biochemical as well as morphological changes. A major one is the induction of COX-2 protein and the subsequent production of PGs (Fig. 1) (33). For these studies, we used KLA, a chemically defined LPS substructure, as it is a single chemical species, highly selective for toll-like receptor 4, and exhibits reproducible endotoxin activity similar to that of native LPS (34). Notably, it was also the agonist used by Lipid Maps for their detailed lipidomic analysis of activated RAW264.7 cells. We focused on changes in eicosanoids produced by the COX-2 pathway at different times following KLA activation. We quantified the levels of PGF_2⍺_, PGE_2_, PGD_2_, and the PGD_2_ dehydration products, PGJ_2_, 15d-PGD_2_, and 15d-PGJ_2_. 6-Keto-PGF_1⍺_, the stable hydrolysis product of PGI_2_, was analyzed but not detected. TxB_2_, which is the hydrolysis product of TxA_2_, was observed, but its levels remained unchanged following KLA treatment. We also observed TxB_2_ in the FBS-containing medium even in the absence of cells.

**Fig. 2:**
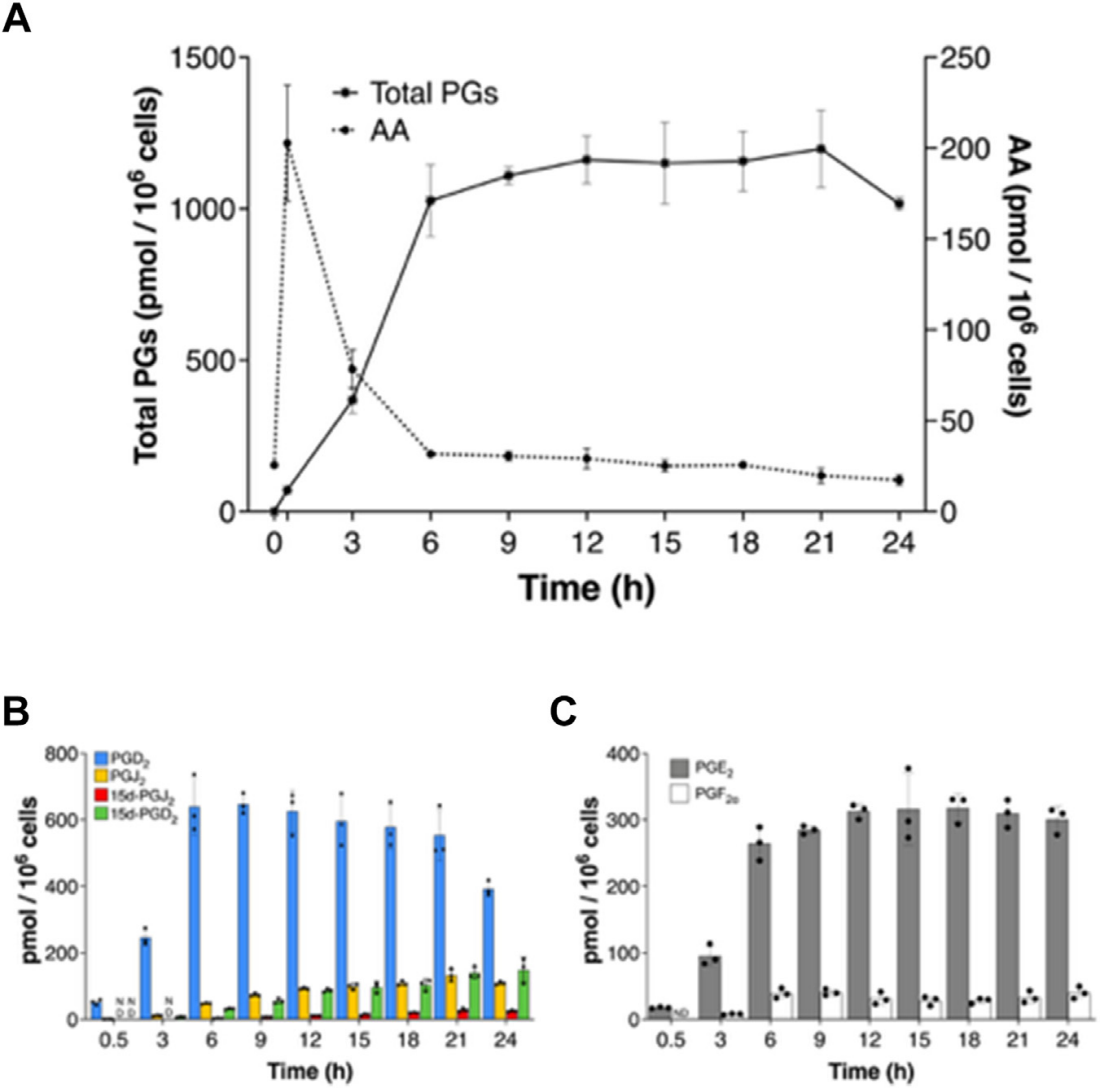
KLA-stimulated AA metabolism in macrophages. RAW264.7 cells were stimulated with 100 ng/ml KLA for the indicated time periods. A: AA and total PGs from 0-24 h. An experiment representing the consistent trend observed over more than 8 experiments is shown, and the data are expressed as mean values ± S.D. of triplicate determinations. B: PGD_2_ and its dehydration products at different times following KLA stimulation. C: PGE_2_ and PGF_2α_ synthesized by RAW264.7 cells at different times following KLA stimulation. AA, arachidonic acid; KLA, Kdo2-lipid A; PG, prostaglandin.

Following KLA stimulation of RAW264.7 cells, there was a dramatic increase in the intracellular levels of AA that peaked at 0.5 h and returned to baseline by 6 h. As the AA levels decreased, PG levels increased and were detected only in the extracellular medium (Fig. 2A). PGD_2_ was the predominant product at all the time points analyzed, followed by PGE_2_ (Fig. 2B, C). PGD_2_ and PGE_2_ were detected as early as 0.5 h after stimulation, and after 3 h of stimulation, the PGD_2_ dehydration products PGJ_2_ and 15d-PGD_2_ were detected. The level of PGD_2_ peaked at 9 h at 650 pmol/10^6^ cells then began to decline slowly such that after 24 h only 394 pmol/10^6^ cells was detected in the medium. The levels of PGJ_2_, 15d-PGJ_2_, and 15d-PGD_2_ in the medium after 24 h were 109, 25, and 149 pmol/10^6^ cells, respectively. The aforementioned values represent the results of the experiment depicted in Fig. 2. The sum of the levels of PGD_2_ and its dehydration products in the medium peaked between 9 and 12 h of activation then remained constant until 24 h (Fig. 2B). The levels of PGE_2_ and PGF_2⍺_ also peaked between 9 and 12 h of activation and stayed constant throughout the remaining incubation period (Fig. 2C).

**Fig. 3:**
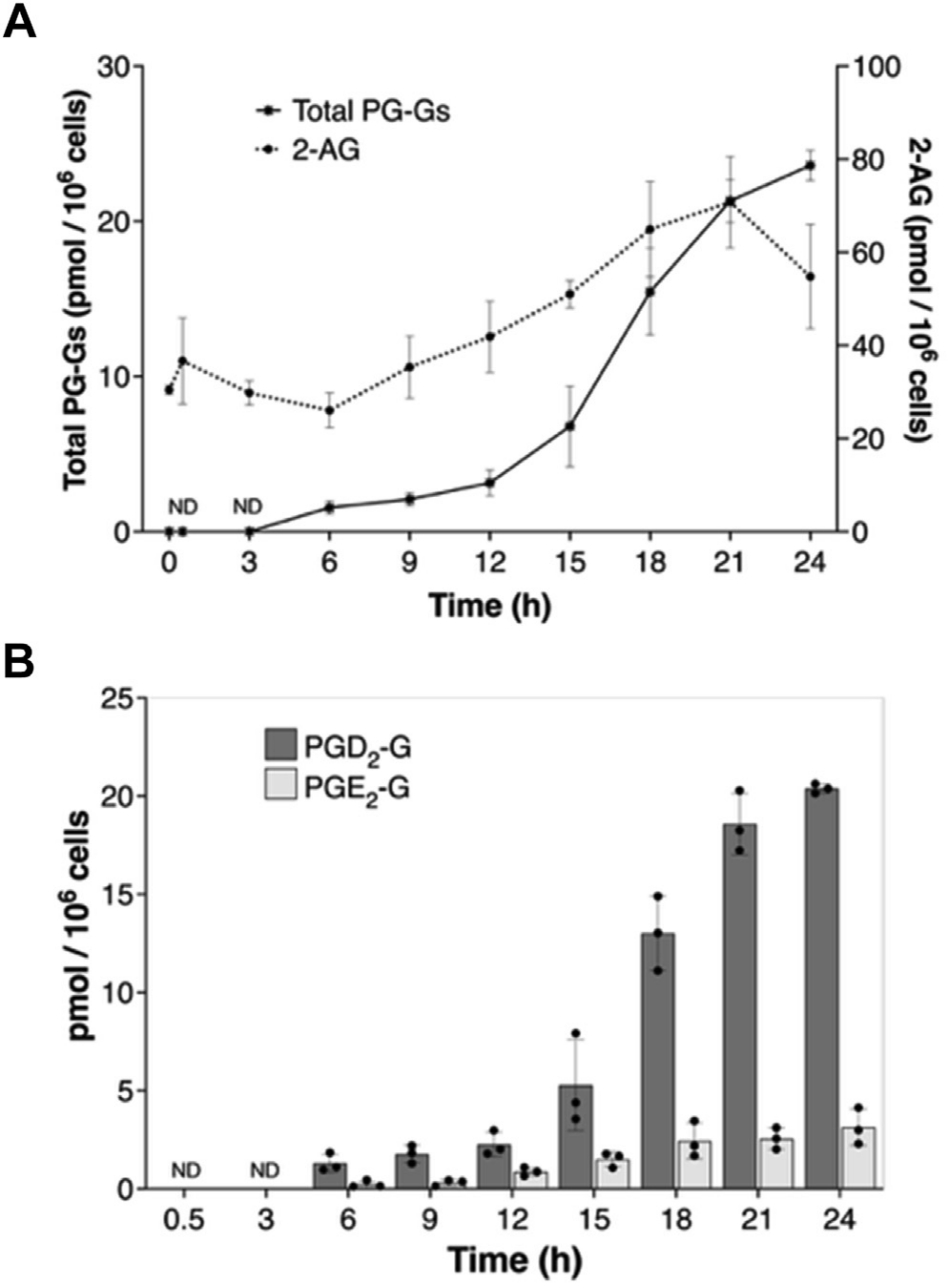
KLA-stimulated 2-AG metabolism in macrophages. RAW264.7 cells were stimulated with 100 ng/ml KLA for the indicated time periods. A: 2-AG and total PG-Gs from 0-24 h. B: Amounts of PGD_2_-G and PGE_2_-G at different times after KLA stimulation. An experiment representing the consistent trend observed over more than 8 experiments is shown, and the data are expressed as mean values ± S.D. of triplicate determinations. ND is not detected. 2-AG, 2-arachidonoylglycerol; KLA, Kdo2-lipid A; PG, prostaglandin; PG-Gs, PG glyceryl esters; PGE2-G, PGE_2_-1-glyceryl ester.

We also quantified the levels of 2-AG and its COX-2 oxygenation products following activation of RAW cells (Fig. 3). During the initial phase of activation (0–12 h), there was no significant change in the level of intracellular 2-AG (Fig. 3A). Minimal levels of PG-Gs were detected until 6 h of stimulation, and after 12 h, only 3 pmol/10^6^ cells of total PG-Gs was present in the medium. However, between 12 and 24 h, there was a significant increase in total PG-G production, while the level of PGs remained constant, as noted above (Figs. 2A and 3A). The increase in PG-Gs correlated to an increase in the levels of intracellular 2-AG, which peaked at 21 h. PGD_2_-G was the major product at all time points with PGE_2_-G the next most abundant product (Fig. 3B). The isomeric composition of the PGD_2_-G and PGE_2_-G was principally the 2-glycerol ester rather than the 1(3)-glycerol ester (supplemental Fig. S3). This corresponded to the observation that the AG was primarily the 2-isomer as opposed to the 1(3)- isomer (supplemental Fig. S4). The levels of PGs were much higher than the levels of PG-Gs at all time points as reported in other macrophage populations (19, 30, 31), but the ratio of PGs/PG-Gs decreased at later time points.

### Hydrolysis of PG-Gs in the cellular milieu

PG-Gs are unstable to hydrolysis, particularly in the presence of esterases. Additionally, the D ring of PGD_2_-G is known to undergo dehydration to a number of products. Thus, we tested PG-G stability in the cellular media used in these studies with and without the presence of RAW cells. Penta-deuterated PGE_2_-G and PGD_2_-G were used in these experiments because the signal from the penta-deuterated compound would not be confounded by endogenously produced PG-Gs. When 300 pmol of PGE_2_-G-d_5_ was added to medium containing KLA-activated RAW264.7 cells, a steady decline in its level was observed such that less than 30% remained after 12 h, and the material was nearly undetectable by 24 h. The half-life for hydrolysis was 8.6 h.

**Fig. 4:**
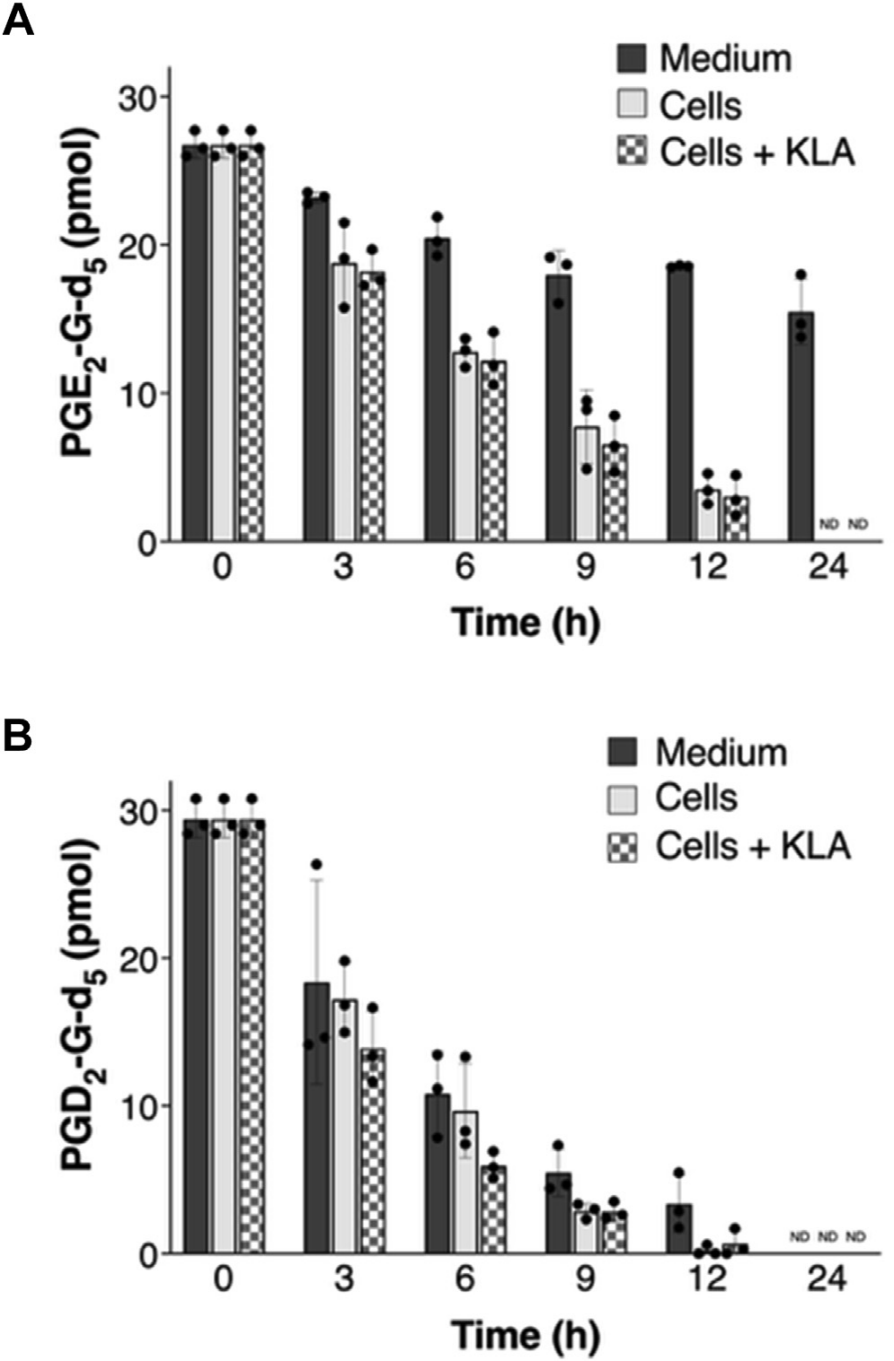
Hydrolysis of PGE_2_-G-d_5_ and PGD_2_-d_5_ A: PGE_2_-G-d_5_ (30 pmol) was added to medium alone, cultures of unactivated RAW264.7 cells, and cultures of KLA-treated RAW264.7 cells. The amount of remaining PGE_2_-G-d_5_ was determined at the indicated time points. Results are the mean values ± S.D. ND is not detected. B: PGD_2_-G-d_5_ (30 pmol) was added to medium alone, cultures of unactivated RAW264.7 cells, and cultures of KLA-treated RAW264.7 cells. The amount of remaining PGD_2_-G-d_5_ was determined at the indicated time points. Results are the mean values ± S.D. ND is not detected. KLA, Kdo2-lipid A; PG, prostaglandin; PGE2-G-d5, PGE_2_-1-glyceryl ester-d_5_.

Parallel experiments were performed in which 30 pmol PGE_2_-G-d_5_ was added to medium alone, medium containing unactivated RAW264.7 cells, or medium containing KLA-activated RAW264.7 cells (Fig. 4A). Although there was a clear decline in the levels of PGE_2_-G-d_5_ in medium alone, the rate of decline was much faster in medium containing cells. Interestingly, there was no difference in the rates of hydrolysis between unactivated and KLA-activated RAW264.7 cells.

We also evaluated the stability of PGD_2_-G-d_5_. PGD_2_-G-d_5_ (30 pmol) was added to medium and medium containing untreated or KLA-treated RAW264.7 cells. PGD_2_-G-d_5_ disappeared 1.7-fold faster than PGE_2_-G-d_5_ in medium alone and medium plus cells (Fig. 4B). There also appeared to be a difference between the rates of disappearance of PGD_2_-G-d_5_ in medium with unactivated cells and medium with KLA-activated cells but this did not achieve statistical significance.

The more rapid disappearance of PGD_2_-G-d_5_ compared to PGE_2_-G-d_5_ may be due to the dehydration of PGD_2_-G-d_5_ which occurs in parallel to hydrolysis; dehydration does not occur with PGE_2_-G-d_5_. However, previous studies have demonstrated that PGD_2_-G is hydrolyzed faster than PGE_2_-G by α/β-hydrolase domain containing 6, which is present in RAW264.7 cells, so differential hydrolysis may play a role as well (35).

These experiments suggest that an appreciable amount of secreted PG-Gs undergo transformation in the medium, and that our quantification at the later stages of the time course, which represent the net of synthesis and hydrolysis, significantly underestimates the amounts of PG-Gs produced by activated RAW264.7 cells. PGD_2_-G is the major product of 2-AG oxidation in RAW264.7 cells and it is more unstable that PGE_2_-G, most likely due to concurrent hydrolysis and dehydration of the D ring. These findings reinforce the conclusion that the total amounts of PG-Gs synthesized by KLA-activated RAW264.7 cells are significantly higher than the steady-state levels measured by mass spectrometry.

### AA-mediated inhibition of 2-AG oxygenation is not responsible for delayed PG-G biosynthesis

**Fig. 5:**
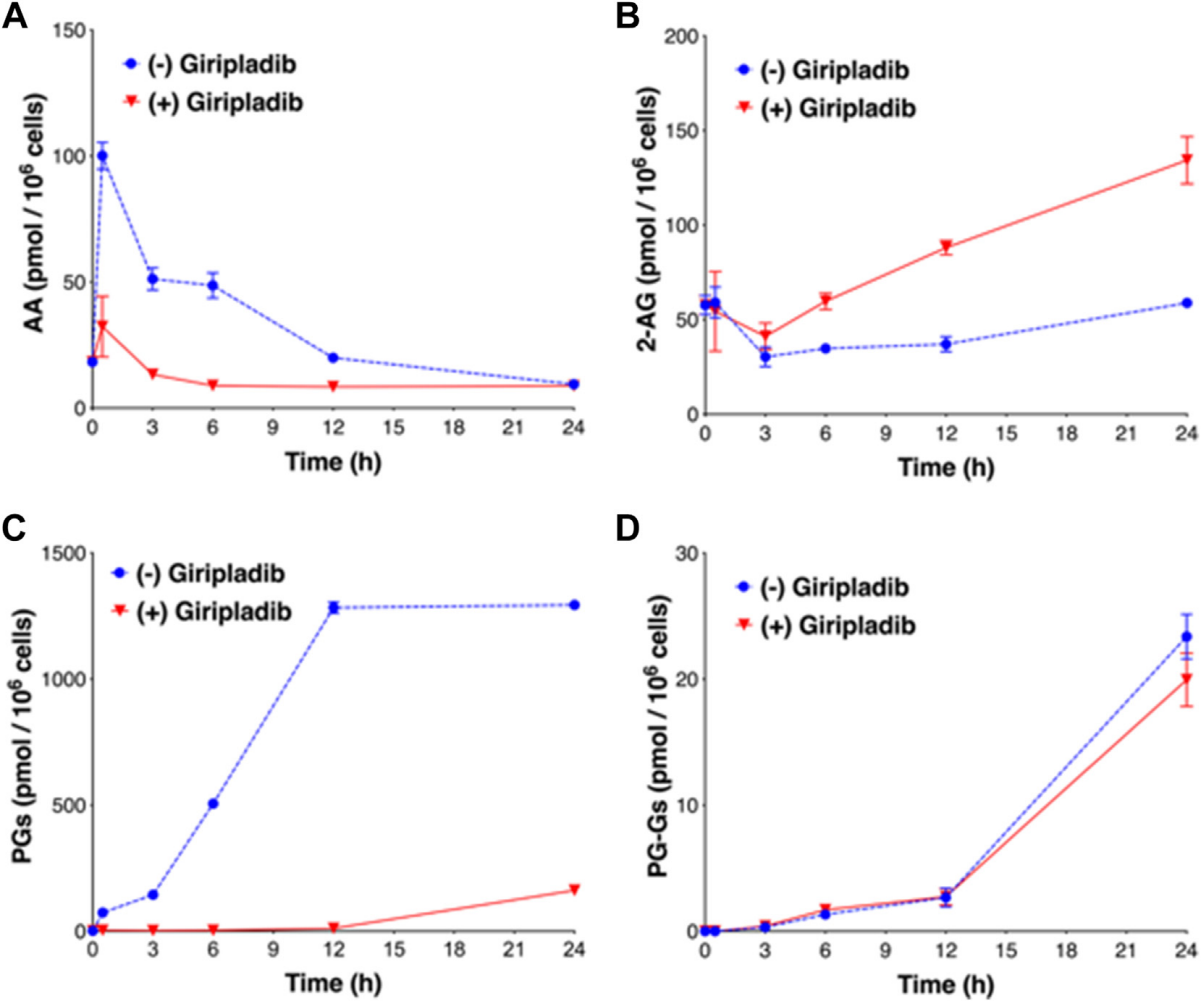
The impact of cPLA2 inhibition on lipid synthesis by RAW264.7 cells. RAW264.7 cells were stimulated with 100 ng/ml KLA in the presence or absence of 100 nM giripladib. The intracellular levels of AA (A) and 2-AG (B), as well as the extracellular levels of PGs (C) and PG-Gs (D) were determined at the indicated time points. Results are the mean values ± S.D. 2-AG, 2-arachidonoylglycerol; AA, arachidonic acid; cPLA2, cytosolic phospholipase A2; KLA, Kdo2-lipid A; PG, prostaglandin; PG-Gs, PG glyceryl esters.

The levels of AA released after KLA treatment were much higher than the levels of 2-AG, and previous studies from our laboratory have demonstrated that AA allosterically inhibits 2-AG oxygenation by COX-2 whereas 2-AG allosterically stimulates AA oxygenation (36). Thus, an obvious hypothesis is that the high level of AA inhibited 2-AG oxygenation during the initial phase of KLA activation of RAW264.7 cells. To test this hypothesis, we used giripladib, a cPLA2 inhibitor, to block release of AA from phospholipids (37). As expected, giripladib addition concomitant with KLA significantly reduced the intracellular levels of AA at all time points analyzed (Fig. 5). Consequently, no PGs were detected during the first 6 h of activation, and levels remained very low throughout the incubation. However, despite the dramatically lower levels of AA at all time points, there was no significant difference in the time course or extent of PG-G formation, implying that AA-mediated inhibition is not responsible for delayed PG-G biosynthesis in RAW264.7 cells. Giripladib treatment had no significant effect on COX-2 or cPLA2 expression (supplemental Fig. S6).

### Time dependence of DAG synthesis and involvement of DAG lipase in 2-AG synthesis during the late phase KLA response

**Fig. 6:**
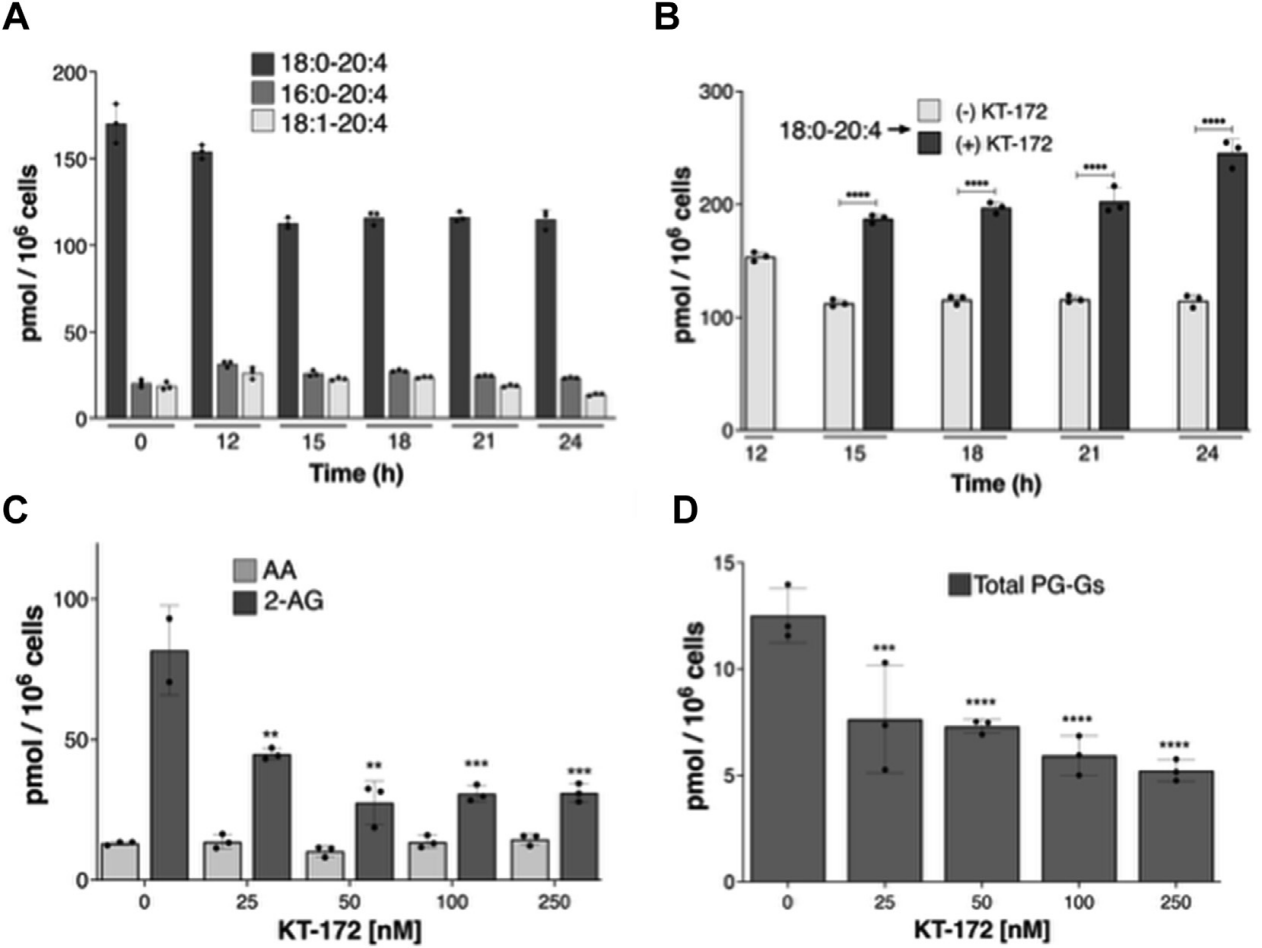
The time dependence of DAG synthesis and its response to KT-172. A: RAW264.7 cells were stimulated with 100 ng/ml KLA for the indicated times, and intracellular levels of AA-containing DAGs were determined at the indicated time points. B: RAW264.7 cells were stimulated with 100 ng/ml KLA, and after 12 h of activation, KT-172 (1 μM) or vehicle was added to the dish. Cells were harvested at the indicated times after KLA addition, and 18:0-20:4 DAG levels were determined. C: RAW264.7 cells were stimulated with 100 ng/ml KLA and after 12 h of activation, different concentrations (25, 50, 100, and 250 nM) of KT-172 were added to the dish. The cells and medium were harvested after 12 h of KT-172 treatment, and intracellular levels of AA and 2-AG were determined. D: Extracellular levels of PG-Gs were determined in the culture medium from cells in the experiment described in C. One-way analysis of variance (ANOVA) was used to determine the statistical significance for KT-172-treated samples relative to the corresponding vehicle-treated controls. ∗*P* < 0.05, ∗∗*P* < 0.001, ∗∗∗*P* value < 0.0005, ∗∗∗∗*P* value < 0.0001. 2-AG, 2-arachidonoylglycerol; AA, arachidonic acid; DAG, diacylglycerol; KLA, Kdo2-lipid A; PG, prostaglandin; PG-Gs, PG glyceryl esters.

Since DAGLα and DAGLβ hydrolyze DAGs to 2-AG (38), the most likely precursor to 2-AG is an arachidonate-containing DAG species. Thus, we determined the intracellular levels of multiple DAG species following KLA activation of RAW264.7 cells (Fig. 6A, B and supplemental Fig. S7). There was a decrease in the level of 18:0-20:4 DAG during the first half of the time course, after which the level remained constant. The other AA-containing DAGs (16:0-20:4 & 18:1-20:4) did not change significantly in response to KLA (Fig. 6A). In contrast, levels of all analyzed saturated or monounsaturated fatty acid-containing DAGs increased (18:1-18:1, 18:0-18:1, 16:0-16:1, 16:0-18:1, 16:1-18:0, and 16:1-18:1, supplemental Fig. S7). The changes in these latter species likely reflect de novo phospholipid biosynthesis via the Kennedy pathway, which generates DAG intermediates comprising predominantly saturated and monounsaturated fatty acids (39).

To probe the role of DAGLs in delayed PG-G synthesis in RAW264.7 cells, we used KT-172, a nonselective inhibitor of both DAG lipase isoforms. The cells were treated with different concentrations of inhibitor following 12 h of activation by KLA. The inhibitor was added after 12 h to minimize the potential for interference in DAG- or 2-AG-dependent signaling events during the early KLA response. As expected, there was no change in the levels of extracellular PGs with any concentration of the compound (supplemental Fig. S8). However, there was a concentration-dependent decrease in both intracellular 2-AG and extracellular PG-G levels (Fig. 6C, D). We also analyzed the intracellular levels of 18:0-20:4 DAG at different times after addition of KT-172 (added after 12 h of stimulation with KLA) and observed a significant increase in this species at 3, 6, 9, and 12 h after addition of the compound (Fig. 6B).

Having established a role for DAG lipases in delayed synthesis of PG-Gs by using a nonselective DAGL inhibitor, we next investigated which isoform may be involved in 2-AG production in RAW264.7 cells. Western blot analyses were performed to determine the expression profiles of DAGLα and DAGLβ at different time points after stimulation with KLA. DAGLα was not detected by Western blot (data not shown), but DAGLβ protein was present in RAW264.7 cells and stably expressed throughout the KLA treatment period (supplemental Fig. S9A). qPCR analysis indicated no change in the levels of DAGLβ mRNA (supplemental Fig. S9B), consistent with the stable levels of protein expression.

## Discussion

Inflammation is an integral part of the body’s defense against pathogens and injury. Upon insult, induction of the acute phase of inflammation and its timely resolution are critical for restoration of homeostasis. Bioactive lipids produced by macrophages play an important role both in the induction and resolution of inflammation. The RAW264.7 cell line has been a valuable model for the study of macrophage responses to microorganisms and their products (32). An extensive body of work exists on the response of RAW264.7 cells to treatment with bacterial LPS or its constituent, KLA (33, 40). However, the available data are lacking a detailed analysis of levels of 2-AG and its COX-2 metabolic products, the PG-Gs.

We discovered that RAW264.7 cells, when stimulated with KLA, produce PGs during the first 12 h of activation followed by a later phase of PG-G biosynthesis (12–24 h). This late-phase PG-G production was associated with a concomitant increase in 2-AG levels. During the early phase of activation, there was a dramatic increase in the intracellular levels of AA after 0.5 h of stimulation that returned to baseline by 6 h. PGD_2_ was the predominant COX-2-generated AA product at all the time points analyzed, followed by PGE_2_. Our results are very similar to those reported in a comprehensive study by Buczynski *et al.*, also in RAW264.7 cells where the AA level peaked in 1 h and returned to basal levels by 4 h (33). In our study, PGD_2_ and PGE_2_ were detected as early as 0.5 h after stimulation whereas the PGD_2_ dehydration products PGJ_2_ and 15d-PGD_2_ could only be detected after 3 h of stimulation. The level of PGD_2_ peaked at 9 h and then began to decline slowly. This decline is attributable to its conversion to PGJ_2_, 15d-PGD_2_, and 15d-PGJ_2_, as their levels increased with time (7, 8). However, the sum of the levels of PGD_2_ and its dehydration metabolites continued to increase over time as was observed for PGE_2_ and PGF_2⍺_, peaking between 9 and 12 h of activation and then remaining constant until 24 h. Buczynski *et al.* observed a similar trend for these analytes (33).

In the present study, the 2-AG oxygenation products, PG-Gs, were not detected until 3 h of stimulation, and levels remained low until 12 h when they began to significantly increase. PG-Gs are also synthesized by the macrophage cell line J774, a well-established model in immunology (19). When stimulated with LPS, these cells synthesize PGD_2_-G at levels of 0.3 pmol/10^6^ cells after 8 h of activation (19). This compares to production of PGD_2_-G by RAW264.7 cells of ∼1 pmol/10^6^ cells at 9 h and ∼20 pmol/10^6^ cells at 24 h following KLA stimulation. It appears that J774 cells are less responsive to LPS than RAW264.7 cells in terms of PG-G synthesis, although the levels of PG-Gs were not measured in J774 cells at time points later than 8 h when the PG-G synthesis by KLA-activated RAW264.7 cells demonstrates a striking increase.

Previous reports from our laboratory indicate that murine resident RPMs pretreated with LPS for 6 h, washed, and then stimulated by zymosan synthesize PG-Gs at levels of ∼2 pmol/10^6^ cells over a period of 2 h, at which time synthesis plateaued (30, 31). Thus, RAW264.7 cells appear to synthesize and release higher amounts of PG-Gs during the late-phase response to KLA than other macrophage models that have been studied to date.

PG-Gs are subject to hydrolysis to PGs and glycerol, and the rate of hydrolysis depends on the availability of esterases that can carry out this reaction. Multiple different enzymes have been reported to hydrolyze PG-Gs including carboxylesterases, monoacylglycerol lipase, α/β-hydrolase domain containing 6 and lysophospholipase A_2_ (35, 41, 42, 43, 44). Considering the lengthy incubation periods used in the present experiments, it was important to consider the impact of hydrolysis on PG-G levels. Indeed, we found that PGE_2_-G is hydrolyzed with a half-life of 8.5 h and is nearly undetectable by 24 h. Although some hydrolysis is observed in FBS-containing medium alone, the rate is higher when cells are present in the medium. There was no difference in the rates of hydrolysis between control and KLA-activated cells. PGD_2_-G disappeared from the medium plus cells even faster (t_1/2_∼3 h) due to the combination of hydrolysis to PGD_2_ and dehydration to PGJ_2_ and related compounds analogous to the PGD_2_ dehydration products shown in supplemental Fig. S1. Given the overlapping time courses for PG-G biosynthesis, hydrolysis, and dehydration, it is apparent that the steady-state levels quantified by LC-MS/MS significantly underestimate the total amount of PG-Gs released by activated RAW264.7 cells. It is possible that differential rates of PG-G hydrolysis contribute to the different amounts quantified following activation of various macrophage populations.

COX-2 oxygenates 2-AG as effectively as AA, and high concentrations of AA inhibit 2-AG oxygenation in vitro (13, 36). KLA stimulates a dramatic increase in AA concentration in RAW264.7 cells shortly after treatment, which raised the possibility that competition with the lower 2-AG concentrations accounted for the lag phase in PG-G production. To test this hypothesis, the cPLA2 inhibitor giripladib was used to block release of AA from phospholipids. As expected, giripladib treatment significantly decreased intracellular AA levels at all time points analyzed, and no PGs were detected during the first 6 h of activation. The PGs generated from 12-24 h in the presence of giripladib may be produced by hydrolysis of PG-Gs, or by hydrolysis of 2-AG to AA followed by COX-2 oxygenation. For example, addition of 2-AG to RPMs leads to rapid hydrolysis to AA and production of PGs at levels 10-fold higher than those of PG-Gs (31). Regardless of the source of the late-stage PGs formed by RAW264.7 cells in the presence of giripladib, no significant difference was observed in the time course or amounts of PG-G formation even though the levels of AA in the cells were dramatically reduced at all time points. This implies that AA-mediated competition is not responsible for delayed PG-G biosynthesis in RAW264.7 cells. A corollary of these observations is that the pool of 2-AG detectable in RAW264.7 cells from 0-6 h is not available to the newly synthesized COX-2 protein.

When RAW264.7 cells were treated with giripladib then stimulated with KLA, the intracellular levels of 2-AG increased (Fig. 5B). A similar result was observed when RPMs from cPLA2^(−/−)^ mice were stimulated with LPS and zymosan (31). The levels of 2-AG nearly doubled compared to those generated by RPMs from WT mice treated with LPS and zymosan. In those experiments, the amounts of PG-Gs synthesized by cPLA2^(−/−)^ mice also increased, which was not observed in the present experiments with RAW264.7 cells (31). RPMs do not hydrolyze PG-Gs (30) whereas RAW264.7 cells do hydrolyze them, so it is possible that differential hydrolysis might contribute to the differences observed with the two cell types. Furthermore, the exact mechanism by which 2-AG levels increase in the presence of cPLA2 blockade is not known. However, it is notable that in the current experiment, KLA-treated RAW264.7 cells produced >1,000 pmol/10^6^ cells of PGs in the absence of giripladib, and in the previous study, zymosan-treated WT RPMs produced ∼2,000 pmol/10^6^ cells of PGs (30, 31). These levels of biosynthesis occur at the expense of AA stores within cellular phospholipids. Thus, it is reasonable to postulate that prevention of AA release by cPLA2 would increase the availability of AA-containing phospholipids that could serve as a source of DAG for 2-AG biosynthesis. Given that 2-AG is a ligand for the CB2 receptor, which is associated with antiinflammatory properties, elevation of 2-AG may contribute to the antiinflammatory effects attributed to cPLA2 inhibitors (37, 45, 46).

Our results indicate that the increase in 2-AG associated with late-stage PG-G biosynthesis in KLA-treated RAW264.7 cells results from hydrolysis of AA-containing DAGs. Thus, the source of those DAG species is of interest. The canonical pathway for the formation of 2-AG via DAG hydrolysis is initiated by the activation of phospholipase C (PLC), which hydrolyzes AA-containing phosphatidylinositol 4,5-bisphosphate (PIP_2_) to DAG. Notably, activation of PLCγ2 has been demonstrated to give rise to 2-AG production in immune complex-activated macrophages (47). Although it is tempting to assume that a PLC-dependent pathway is active in late-stage PG-G biosynthesis, the vast majority of prior studies of the role of PLC in signaling pathways indicate that its activation and production of DAG occurs over a time course of less than 30 min. PLCε has been reported to undergo sustained activation, but even under those circumstances, the production of DAG has only been observed for a period of up to 1 h (14, 48, 49).

A second consideration regarding the possible role of PLC in PG-G biosynthesis involves the availability of adequate quantities of substrate. PIP_2_ the canonical PLC substrate is present in minor quantities, primarily in the plasma membrane. If PIP_2_ serves as the immediate precursor for DAGs utilized for PG-G synthesis in KLA-treated RAW264.7 cells, then an ongoing regeneration from PI would likely be required. Alternatively, one could postulate that under some circumstances, PLC could utilize PIP or PI as a substrate, as has been demonstrated for some isoforms in vitro (50, 51, 52, 53). In this regard, it is interesting that data from the Lipid Maps consortium reveal an absolute decrease in the quantity of AA-containing PIs during the KLA response. In fact, the only AA-containing glycerophospholipids that undergo a sustained quantitative decrease are PIs (40). These considerations suggest that, if a PLC is responsible for DAG formation during late-phase PG-G synthesis, it would represent a novel function for this enzyme, both in terms of the time course of activation and, possibly, the preferred substrate. Nevertheless, we tested the hypothesis that a PLC could be involved by evaluating the effects of the pan-PLC inhibitor U73122 on PG-G synthesis in RAW264.7 cells. Although an inhibition of late-stage PG-G biosynthesis was observed in the presence of U73122, a similar result was obtained with the negative control compound U73343, suggesting a nonspecific effect. Together, these findings argue against the involvement of a PLC in late-stage DAG production; however, there are many isoforms of PLC, and the specificity and mechanism of U73122 is in question. Thus, we cannot presently rule out the possibility that a PLC isoform undergoes prolonged activation leading to the observed elevated DAG levels during the latter phases of the KLA response (49).

An alternative pathway for the generation of DAG involves phospholipase D (PLD)-dependent hydrolysis of phosphatidyl choline to phosphatidic acid which can then be hydrolyzed to DAG. In the current experiments, no significant change in 2-AG or PG-Gs was observed upon incubation of the RAW264.7 cells with KLA in the presence of a nonspecific (ML-299) or isoform-specific inhibitors of PLDs (VU0155069 for PLD1 and ML-298 for PLD2) (data not shown). Similarly, the nonspecific phosphatidic acid phosphatase inhibitor propranolol had no effect on PG-G biosynthesis in KLA-treated RAW264.7 cells.

Recently, another pathway for the conversion of AA to 2-AG has been reported in human leukocytes. This pathway involves the incorporation of AA into cellular phospholipids followed by hydrolysis to release precursors of 2-AG; lysophosphatidic acid may be an intermediate (54). Given the burst of AA release following KLA treatment, it seemed possible that the AA could be recycled into this other pathway for the delayed generation of 2-AG. However, if such a pathway was responsible for 2-AG production in KLA-activated RAW264.7 cells, blocking the release of AA with the cPLA2 inhibitor, giripladib, should block subsequent production of 2-AG. This was not the case, so it appears that 2-AG is not produced by this novel pathway in RAW264.7 cells.

Another pathway to be considered as a potential source of late-stage 2-AG is the direct acylation of glycerol by arachidonoyl-coenzyme A or transacylation of glycerol from a phospholipid. Previous studies have shown that AA can be incorporated by rat myoblast or liver cell lines into arachidonoylglycerol, presumably via the intermediacy of the coenzyme A derivative (55). Transacylation from phospholipid to glycerol can be catalyzed by purified cPLA2 or lysophospholipase-transacylase (56, 57). The initial product in all three cases is 1(3)-AG. The AG released following KLA activation is the 2-isomer, 2-AG, and the PG-Gs are predominantly the 2-glycerol esters. Furthermore, arachidonoyl acylation of glycerol in intact cells is much less efficient than arachidonoyl acylation of glycerol phosphate and is only significant when glycerol phosphate synthesis is inhibited. Therefore, it seems unlikely that the burst of 2-AG that we detect in RAW cells between 12-24 h is due to direct acylation or transacylation of glycerol.

Conversely, it is possible that the increase in 2-AG levels in KLA-activated RAW264.7 cells results from inhibition of its hydrolysis somehow affected by KLA treatment. We tested this possibility by comparing the rates of hydrolysis of 2-AG-d_5_ in medium or medium containing unactivated or KLA-activated RAW264.7 cells. All incubations were conducted in the presence of indomethacin to prevent 2-AG oxidation by COX-2 induced by KLA treatment. The time course for disappearance of 2-AG-d_5_ was faster in the presence of cells than in their absence, but there was no difference in the time courses for hydrolysis of 2-AG-d_5_ between unactivated and KLA-activated RAW264.7 cells (Supplemental Fig. S10). Thus, differential hydrolysis does not account for the increase in 2-AG levels stimulated by KLA.

DAGLβ, but not DAGLα, is reported to exist in RAW264.7 cells and other macrophage populations (58). Consistent with these previous reports, we detected DAGLβ but not DAGLα by Western blot in our experiments. To test the role of DAGLβ in delayed PG-G synthesis in RAW264.7 cells, we used KT-172, a nonselective inhibitor of both DAGL isoforms (58). The cells were treated with different concentrations of inhibitor added after 12 h of activation, which resulted in a concentration-dependent decrease in both intracellular 2-AG and extracellular PG-G levels. In addition, there was a significant increase in the intracellular level of 18:0-20:4 DAG at all the time points analyzed, similar to previous findings in thioglycolate-elicited peritoneal and LPS-activated peritoneal macrophages treated with DAGLβ inhibitors. No significant change in the levels of PGs or AA was seen at the inhibitor concentrations used. This result suggests that DAGLβ is involved in late phase 2-AG metabolism.

**Fig. 7:**
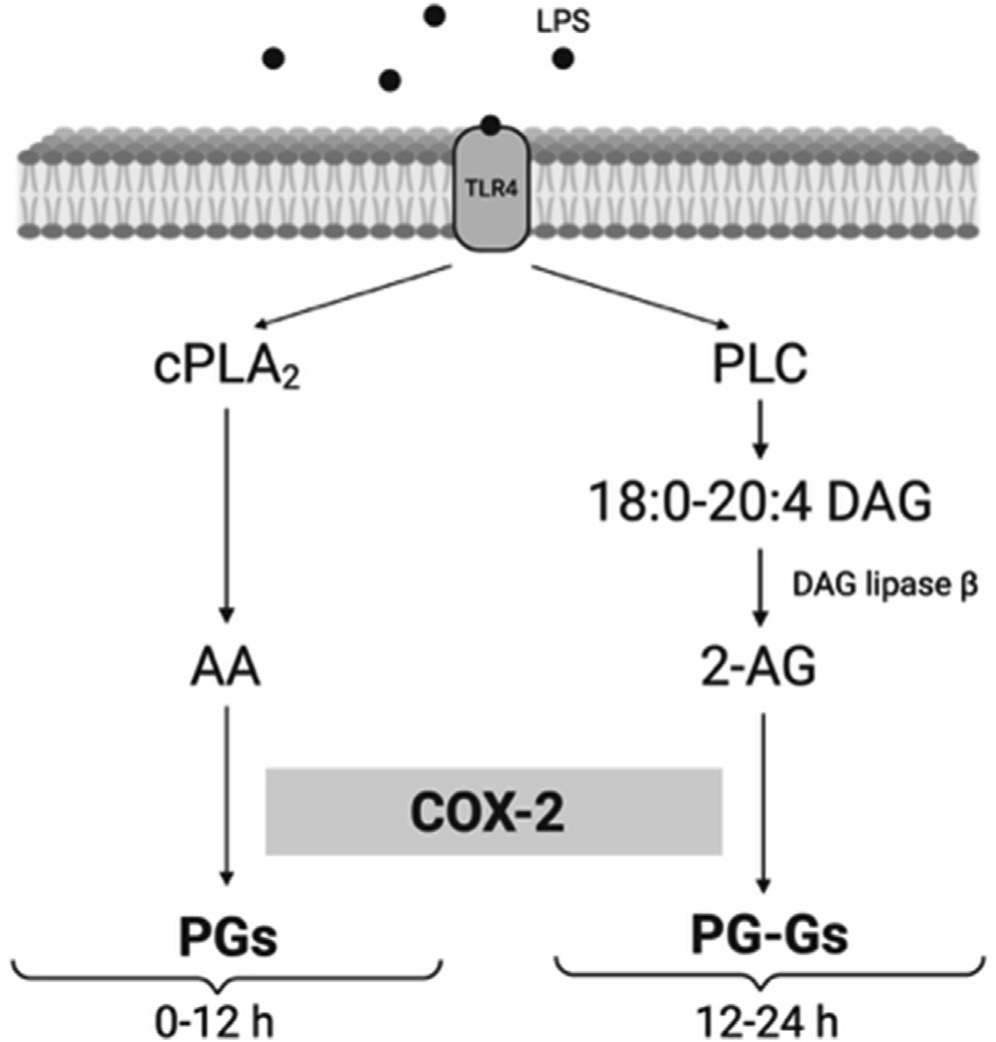
Summary of the generation and oxidation of AA and 2-AG by KLA-activated RAW264.7 cells. 2-AG, 2-arachidonoylglycerol; AA, arachidonic acid; KLA, Kdo2-lipid A.

Figure 7 summarizes our findings on the production of PGs and PG-Gs in activated RAW264.7 cells. As others have reported, the initial burst of AA produced by the action of cPLA2 is followed by AA oxygenation to PGs by newly synthesized COX-2 and by its reincorporation into phospholipids. The massive production of PGs is complete by 9 h although PGD_2_ continues to be converted nonenzymatically to a series of dehydration products. As PG production plateaus, DAG is converted to 2-AG by the action of DAGLβ. This pool of 2-AG is oxidized by COX-2 to form PG-Gs. PG-G production is maximal by 24 h.

Several aspects are worth noting about the levels of PG-Gs that we quantified by LC-MS/MS. First, RAW264.7 cells are impressive producers of PG-Gs compared to other macrophage cell lines for which data have been reported. For example, the levels of PGD_2_-G generated by RAW264.7 cells are higher than the levels reported in the J774 cell line and in murine RPMs (19). In fact, the levels in RAW264.7 cell culture medium represent an underestimate of the amounts released by the cells due to the hydrolysis of PG-Gs to PGs. A similar fact may complicate the levels reported for other macrophage cell lines although RPMs do not significantly hydrolyze PG-Gs during short-term incubations of 2 h (30, 31). Second, the levels of PGs are much higher than those of PG-Gs, especially in the first 9–12 h after KLA treatment. To some extent, this reflects the difference in pool sizes between AA and 2-AG. The maximum level of AA is 10-20-fold higher than the maximum level of 2-AG. It also appears that AA is oxygenated more readily than 2-AG by COX-2 in cells. For example, only the second pool of 2-AG released following KLA treatment is oxygenated by COX-2. Finally, although the levels of PG-Gs are much lower than the levels of PGs, the levels of PG-Gs are biologically significant. The levels of PGE_2_-G quantified after activation of RAW264.7 cells are ∼2–5 pmol/10^6^ cells, which corresponds to 1–2 nM in the culture medium. This is 1,000-fold higher than the concentrations that activate Ca^2+^ mobilization in RAW264.7 cells and that activate the P2Y_6_ receptor when expressed in HEK293 cells (16, 18, 22). Furthermore, Alhouayek *et al.* have reported that PGD_2_-G produced at endogenous levels by J774 cells downregulates interleukin-1β production by the macrophages (19). As noted above, the levels of PGD_2_-G generated by RAW264.7 cells are significantly higher than the levels produced by J774 cells.

Prior investigations of lipid metabolism in RAW264.7 macrophages did not address the production of the endocannabinoid, 2-AG, or its oxygenation products via the COX pathway. The present study reveals that these cells generate significant amounts of 2-AG with a time course that is delayed by nearly 12 h from the generation of AA. The pathway for 2-AG generation appears to involve hydrolysis of AA-containing DAG by DAGLβ. The released 2-AG is then oxygenated by COX-2 to PGH_2_-G, which is converted primarily to PGD_2_-G (major) and PGE_2_-G (minor). The biological activities of 2-AG and the PG-Gs suggest that they may play important roles as either antiinflammatory or proinflammatory mediators. It will be interesting to define the molecular events responsible for the distinctive time course of endocannabinoid metabolism in RAW264.7 cells and their relevance to the control of inflammation.

## Data availability

All data are available in this manuscript.

## Supplemental data

This article contains supplemental data (59).

## Conflict of interest

The authors declare that they have no conflicts of interest with the contents of this article.

## References

[1] Hajeyah A.A., Griffiths W.J., Wang Y., Finch A.J., O'Donnell V.B. (2020). The biosynthesis of enzymatically oxidized lipids. Front. Endocrinol. (Lausanne).

[2] Zhang Y., Liu Y., Sun J., Zhang W., Guo Z., Ma Q. (2023). Arachidonic acid metabolism in health and disease. MedComm (2020).

[3] Smith W.L., Marnett L.J. (1991). Prostaglandin endoperoxide synthase: structure and catalysis. Biochim. Biophys. Acta.

[4] Sun F.F., Chapman J.P., McGuire J.C. (1977). Metabolism of prostaglandin endoperoxide in animal tissues. Prostaglandins.

[5] Samuelsson B., Goldyne M., Granström E., Hamberg M., Hammarström S., Malmsten C. (1978). Prostaglandins and thromboxanes. Annu. Rev. Biochem..

[6] Hata A.N., Breyer R.M. (2004). Pharmacology and signaling of prostaglandin receptors: multiple roles in inflammation and immune modulation. Pharmacol. Ther..

[7] Fitzpatrick F.A., Wynalda M.A. (1983). Albumin-catalyzed metabolism of prostaglandin D2. Identification of products formed in vitro. J. Biol. Chem..

[8] Kikawa Y., Narumiya S., Fukushima M., Wakatsuka H., Hayaishi O. (1984). 9-Deoxy-delta 9, delta 12-13,14-dihydroprostaglandin D2, a metabolite of prostaglandin D2 formed in human plasma. Proc. Natl. Acad. Sci. U. S. A..

[9] Kliewer S.A., Lenhard J.M., Willson T.M., Patel I., Morris D.C., Lehmann J.M. (1995). A prostaglandin J2 metabolite binds peroxisome proliferator-activated receptor gamma and promotes adipocyte differentiation. Cell.

[10] Lands W.E., Samuelsson B. (1968). Phospholipid precursors of prostaglandins. Biochim. Biophys. Acta.

[11] Mouchlis V.D., Dennis E.A. (2019). Phospholipase A(2) catalysis and lipid mediator lipidomics. Biochim. Biophys. Acta Mol. Cell Biol. Lipids.

[12] Yu M., Ives D., Ramesha C.S. (1997). Synthesis of prostaglandin E2 ethanolamide from anandamide by cyclooxygenase-2. J. Biol. Chem..

[13] Kozak K.R., Rowlinson S.W., Marnett L.J. (2000). Oxygenation of the endocannabinoid, 2-arachidonylglycerol, to glyceryl prostaglandins by cyclooxygenase-2. J. Biol. Chem..

[14] Smrcka A.V., Brown J.H., Holz G.G. (2012). Role of phospholipase Cepsilon in physiological phosphoinositide signaling networks. Cell Signal..

[15] Kozak K.R., Crews B.C., Morrow J.D., Wang L.H., Ma Y.H., Weinander R. (2002). Metabolism of the endocannabinoids, 2-arachidonylglycerol and anandamide, into prostaglandin, thromboxane, and prostacyclin glycerol esters and ethanolamides. J. Biol. Chem..

[16] Nirodi C.S., Crews B.C., Kozak K.R., Morrow J.D., Marnett L.J. (2004). The glyceryl ester of prostaglandin E2 mobilizes calcium and activates signal transduction in RAW264.7 cells. Proc. Natl. Acad. Sci. U. S. A..

[17] Sang N., Zhang J., Marcheselli V., Bazan N.G., Chen C. (2005). Postsynaptically synthesized prostaglandin E2 (PGE2) modulates hippocampal synaptic transmission via a presynaptic PGE2 EP2 receptor. J. Neurosci..

[18] Richie-Jannetta R., Nirodi C.S., Crews B.C., Woodward D.F., Wang J.W., Duff P.T., Marnett L.J. (2010). Structural determinants for calcium mobilization by prostaglandin E2 and prostaglandin F2alpha glyceryl esters in RAW 264.7 cells and H1819 cells. Prostaglandins Other Lipid Mediat..

[19] Alhouayek M., Masquelier J., Cani P.D., Lambert D.M., Muccioli G.G. (2013). Implication of the anti-inflammatory bioactive lipid prostaglandin D2-glycerol ester in the control of macrophage activation and inflammation by ABHD6. Proc. Natl. Acad. Sci. U. S. A..

[20] Alhouayek M., Buisseret B., Paquot A., Guillemot-Legris O., Muccioli G.G. (2018). The endogenous bioactive lipid prostaglandin D(2)-glycerol ester reduces murine colitis via DP1 and PPARgamma receptors. FASEB J..

[21] Khasabova I.A., Uhelski M., Khasabov S.G., Gupta K., Seybold V.S., Simone D.A. (2019). Sensitization of nociceptors by prostaglandin E(2)-glycerol contributes to hyperalgesia in mice with sickle cell disease. Blood.

[22] Bruser A., Zimmermann A., Crews B.C., Sliwoski G., Meiler J., König G.M. (2017). Prostaglandin E(2) glyceryl ester is an endogenous agonist of the nucleotide receptor P2Y(6). Sci. Rep..

[23] Baggelaar M.P., Maccarrone M., van der Stelt M. (2018). 2-Arachidonoylglycerol: a signaling lipid with manifold actions in the brain. Prog. Lipid Res..

[24] Ferreira S.H., Moncada S., Vane J.R. (1971). Indomethacin and aspirin abolish prostaglandin release from the spleen. Nat. New Biol..

[25] Smith J.B., Willis A.L. (1971). Aspirin selectively inhibits prostaglandin production in human platelets. Nat. New Biol..

[26] Picot D., Loll P.J., Garavito R.M. (1994). The X-ray crystal structure of the membrane protein prostaglandin H2 synthase-1. Nature.

[27] Kurumbail R.G., Stevens A.M., Gierse J.K., McDonald J.J., Stegeman R.A., Pak J.Y. (1996). Structural basis for selective inhibition of cyclooxygenase-2 by anti-inflammatory agents. Nature.

[28] Warner T.D., Mitchell J.A. (2004). Cyclooxygenases: new forms, new inhibitors, and lessons from the clinic. FASEB J..

[29] Rouzer C.A., Marnett L.J. (2020). Structural and chemical biology of the interaction of cyclooxygenase with substrates and non-steroidal anti-inflammatory drugs. Chem. Rev..

[30] Rouzer C.A., Marnett L.J. (2005). Glycerylprostaglandin synthesis by resident peritoneal macrophages in response to a zymosan stimulus. J. Biol. Chem..

[31] Rouzer C.A., Tranguch S., Wang H., Zhang H., Dey S.K., Marnett L.J. (2006). Zymosan-induced glycerylprostaglandin and prostaglandin synthesis in resident peritoneal macrophages: roles of cyclo-oxygenase-1 and -2. Biochem. J..

[32] Raschke W.C., Baird S., Ralph P., Nakoinz I. (1978). Functional macrophage cell lines transformed by Abelson leukemia virus. Cell.

[33] Buczynski M.W., Stephens D.L., Bowers-Gentry R.C., Grkovich A., Deems R.A., Dennis E.A. (2007). TLR-4 and sustained calcium agonists synergistically produce eicosanoids independent of protein synthesis in RAW264.7 cells. J. Biol. Chem..

[34] Raetz C.R., Garrett T.A., Reynolds C.M., Shaw W.A., Moore J.D., Smith D.C. (2006). Kdo2-Lipid A of Escherichia coli, a defined endotoxin that activates macrophages via TLR-4. J. Lipid Res..

[35] Savinainen J.R., Kansanen E., Pantsar T., Navia-Paldanius D., Parkkari T., Lehtonen M. (2014). Robust hydrolysis of prostaglandin glycerol esters by human monoacylglycerol lipase (MAGL). Mol. Pharmacol..

[36] Mitchener M.M., Hermanson D.J., Shockley E.M., Brown H.A., Lindsley C.W., Reese J. (2015). Competition and allostery govern substrate selectivity of cyclooxygenase-2. Proc. Natl. Acad. Sci. U. S. A..

[37] Lee K.L., Foley M.A., Chen L., Behnke M.L., Lovering F.E., Kirincich S.J. (2007). Discovery of Ecopladib, an indole inhibitor of cytosolic phospholipase A2alpha. J. Med. Chem..

[38] Reisenberg M., Singh P.K., Williams G., Doherty P. (2012). The diacylglycerol lipases: structure, regulation and roles in and beyond endocannabinoid signalling. Philos. Trans. R. Soc. Lond. B Biol. Sci..

[39] Yamashita A., Hayashi Y., Nemoto-Sasaki Y., Ito M., Oka S., Tanikawa T. (2014). Acyltransferases and transacylases that determine the fatty acid composition of glycerolipids and the metabolism of bioactive lipid mediators in mammalian cells and model organisms. Prog. Lipid Res..

[40] Dennis E.A., Deems R.A., Harkewicz R., Quehenberger O., Brown H.A., Milne S.B. (2010). A mouse macrophage lipidome. J. Biol. Chem..

[41] Kozak K.R., Crews B.C., Ray J.L., Tai H.H., Morrow J.D., Marnett L.J. (2001). Metabolism of prostaglandin glycerol esters and prostaglandin ethanolamides in vitro and in vivo. J. Biol. Chem..

[42] Xie S., Borazjani A., Hatfield M.J., Edwards C.C., Potter P.M., Ross M.K. (2010). Inactivation of lipid glyceryl ester metabolism in human THP1 monocytes/macrophages by activated organophosphorus insecticides: role of carboxylesterases 1 and 2. Chem. Res. Toxicol..

[43] Manna J.D., Wepy J.A., Hsu K.L., Chang J.W., Cravatt B.F., Marnett L.J. (2014). Identification of the major prostaglandin glycerol ester hydrolase in human cancer cells. J. Biol. Chem..

[44] Turcotte C., Dumais É., Archambault A.S., Martin C., Blanchet M.R., Bissonnette É. (2019). Human leukocytes differentially express endocannabinoid-glycerol lipases and hydrolyze 2-arachidonoyl-glycerol and its metabolites from the 15-lipoxygenase and cyclooxygenase pathways. J. Leukoc. Biol..

[45] Balsinde J., Balboa M.A., Insel P.A., Dennis E.A. (1999). Regulation and inhibition of phospholipase A2. Annu. Rev. Pharmacol. Toxicol..

[46] Nikolaou A., Kokotou M.G., Vasilakaki S., Kokotos G. (2019). Small-molecule inhibitors as potential therapeutics and as tools to understand the role of phospholipases A(2). Biochim. Biophys. Acta Mol. Cell Biol. Lipids.

[47] Jing H., Reed A., Ulanovskaya O.A., Grigoleit J.S., Herbst D.M., Henry C.L. (2021). Phospholipase Cgamma2 regulates endocannabinoid and eicosanoid networks in innate immune cells. Proc. Natl. Acad. Sci. U. S. A..

[48] Dusaban S.S., Brown J.H. (2015). PLCepsilon mediated sustained signaling pathways. Adv. Biol. Regul..

[49] Katan M., Cockcroft S. (2020). Phospholipase C families: common themes and versatility in physiology and pathology. Prog. Lipid Res..

[50] Banno Y., Yu A., Nakashima T., Homma Y., Takenawa T., Nozawa Y. (1990). Purification and characterization of a cytosolic phosphoinositide-phospholipase C (gamma 2-type) from human platelets. Biochem. Biophys. Res. Commun..

[51] Rhee S.G., Choi K.D. (1992). Regulation of inositol phospholipid-specific phospholipase C isozymes. J. Biol. Chem..

[52] Ellis M.V., James S.R., Perisic O., Downes C.P., Williams R.L., Katan M. (1998). Catalytic domain of phosphoinositide-specific phospholipase C (PLC). Mutational analysis of residues within the active site and hydrophobic ridge of plcdelta1. J. Biol. Chem..

[53] Seifert J.P., Wing M.R., Snyder J.T., Gershburg S., Sondek J., Harden T.K. (2004). RhoA activates purified phospholipase C-epsilon by a guanine nucleotide-dependent mechanism. J. Biol. Chem..

[54] Turcotte C., Archambault A.S., Dumais É., Martin C., Blanchet M.R., Bissonnette E. (2020). Endocannabinoid hydrolysis inhibition unmasks that unsaturated fatty acids induce a robust biosynthesis of 2-arachidonoyl-glycerol and its congeners in human myeloid leukocytes. FASEB J..

[55] Lee D.P., Deonarine A.S., Kienetz M., Zhu Q., Skrzypczak M., Chan M., Choy P.C. (2001). A novel pathway for lipid biosynthesis: the direct acylation of glycerol. J. Lipid Res..

[56] Hanel A.M., Gelb M.H. (1995). Multiple enzymatic activities of the human cytosolic 85-kDa phospholipase A2: hydrolytic reactions and acyl transfer to glycerol. Biochemistry.

[57] Sugimoto H., Yamashita S. (1999). Characterization of the transacylase activity of rat liver 60-kDa lysophospholipase-transacylase. Acyl transfer from the sn-2 to the sn-1 position. Biochim. Biophys. Acta.

[58] Hsu K.L., Tsuboi K., Adibekian A., Pugh H., Masuda K., Cravatt B.F. (2012). DAGLbeta inhibition perturbs a lipid network involved in macrophage inflammatory responses. Nat. Chem. Biol..

[59] Rouzer C.A., Ghebreselasie K., Marnett L.J. (2002). Chemical stability of 2-arachidonoylglycerol under biological conditions. Chem. Phys. Lipids.

