# Supplementary material for: Temporal dissociation of COX-2-dependent arachidonic acid and 2-arachidonoylglycerol metabolism in RAW264.7 macrophages
Source: J Lipid Res. 2024 Aug 5;65(9):100615. doi: 10.1016/j.jlr.2024.100615 (PMC11401187; doi:10.1016/j.jlr.2024.100615)
Supplement: Supplemental Data [file mmc1.pdf]

Temporal Dissociation of COX-2-Dependent Arachidonic Acid and 2-Arachidonoylglycerol Metabolism in RAW264.7 Macrophages

Ansari M. Aleem[†], Michelle M. Mitchener[†], Philip J. Kingsley, Carol A. Rouzer, and Lawrence J. Marnett[*]

A. B. Hancock, Jr., Memorial Laboratory for Cancer Research, Departments of Biochemistry, Chemistry and Pharmacology, Vanderbilt Institute of Chemical Biology, and Vanderbilt-Ingram Cancer Center, Vanderbilt University School of Medicine, Nashville, Tennessee 37232, USA

Supplemental Data

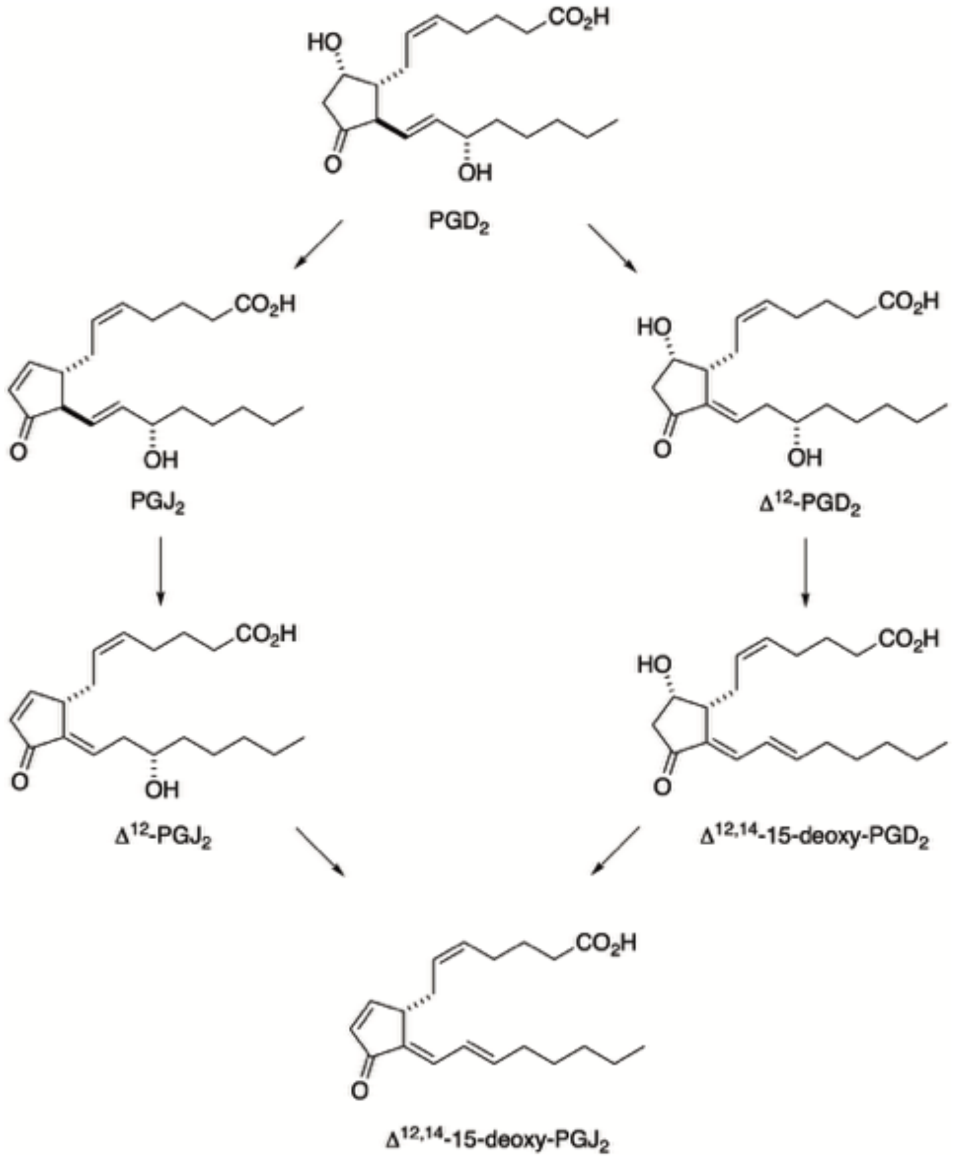

Fig. S1. PGD_2 and its dehydration/rearrangement products.

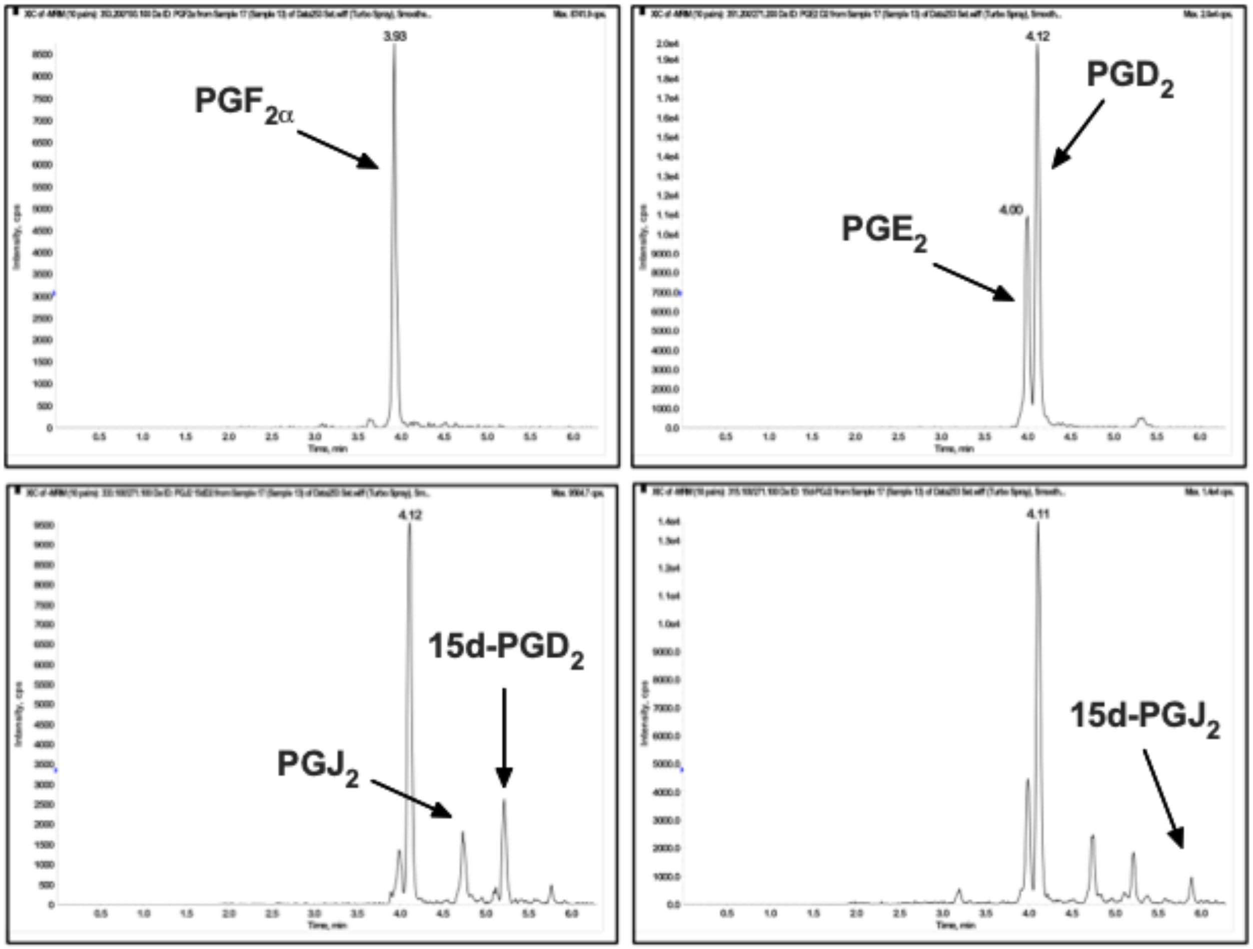

Fig. S2. LC-MS/MS chromatograms of PG species: These representative chromatograms were obtained from analysis of cell medium collected from a plate of 10^6 RAW264.7 cells after 24 h of activation as described in Materials and Methods. Extracted PG species were analyzed on the described LC-MS/MS system in negative ion mode. The specific MRM transitions are given in Table S1, and specific chromatographic parameters are given in the table below.

**Parameter**	**Value**
Mobile Phase A	H_2O + 0.1% formic acid
Mobile Phase B	Acetonitrile:methanol (3:1) + 0.1% formic acid
Column	Phenomenex Luna(2) C18 5 x 0.2 cm, 3 um @ 43C
Gradient	25% B to 72% B in 4.5 min

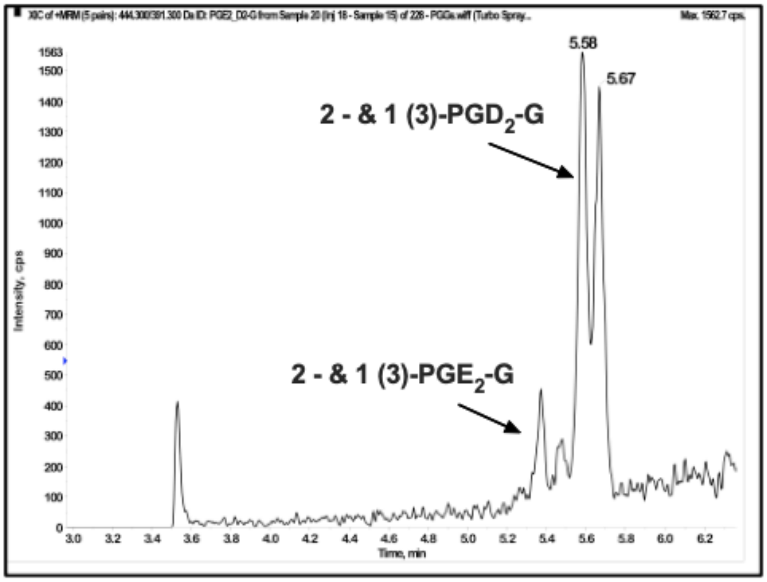

Fig. S3. LC-MS/MS chromatograms of PG-G species: These representative chromatograms were obtained from analysis of cell medium collected from a plate of 10^6 RAW264.7 cells after 24 h of activation as described in Materials and Methods. Extracted PG-Gs species were analyzed on the described LC-MS/MS system in positive ion mode. The specific MRM transitions are given in Table S1, and specific chromatographic parameters are given in the table below.

Monoacylglycerols exist in an equilibrium between the 1(3)- and 2- isomer [1]. The 2-isomer is the initial product, and the 1(3)- isomer is a product of acyl migration. Under the analytical conditions described here, the 2-isomer elutes first, followed by the 1(3)-isomer. While there is considerable chromatographic resolution between these 2 isomeric forms, our group integrates both peaks together.

**Parameter**	**Value**
Mobile Phase A	H_2O with 10 mM ammonium acetate, pH'd to ~3.6
Mobile Phase B	Acetonitrile with ~15% mobile phase A
Column	Phenomenex Luna(2) C18 10 x 0.2 cm, 3 um @ 43C
Gradient	25%B to 65% B in 6 min

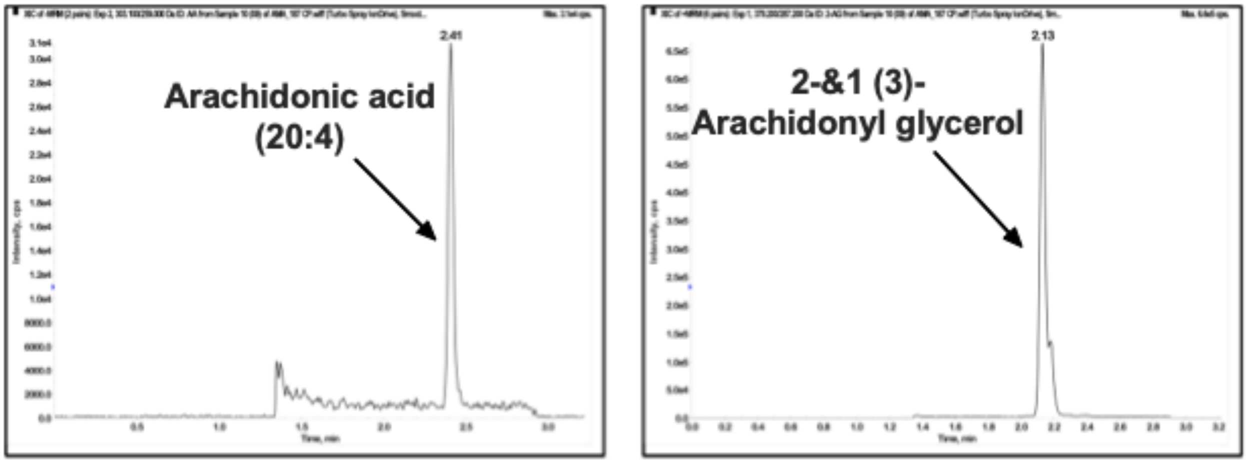

Fig. S4. LC-MS/MS chromatograms of arachidonic acid and arachidonoylglycerol isomers: These representative chromatograms were obtained from analysis of a cell pellet collected from a plate of 10^6 RAW264.7 cells after 24 h of activation as described in Materials and Methods. Arachidonic acid and arachidonoylglycerol isomers were analyzed on the described LC-MS/MS system in negative and positive ion mode, respectively. The specific MRM transitions are given in Table S1, and specific chromatographic parameters are given in the table below.

Similar to PG-Gs discussed in Fig. S3, arachidonoylglycerol exists as both the 1(3)- and 2- isomers, with the 2-isomer eluting first. The two isomers, which are not fully resolved, are integrated together. Thus, the 2-AG levels reported contain a minor component of 1(3)-AG.

**Parameter**	**Value**
Mobile Phase A	H_2O + 0.1% formic acid
Mobile Phase B	Acetonitrile:methanol (3:1) + 0.1% formic acid
Column	Acquity C18 5 x 0.2 cm, 1.8 um @ 43C
Gradient	65%B to 100% B in 3 min

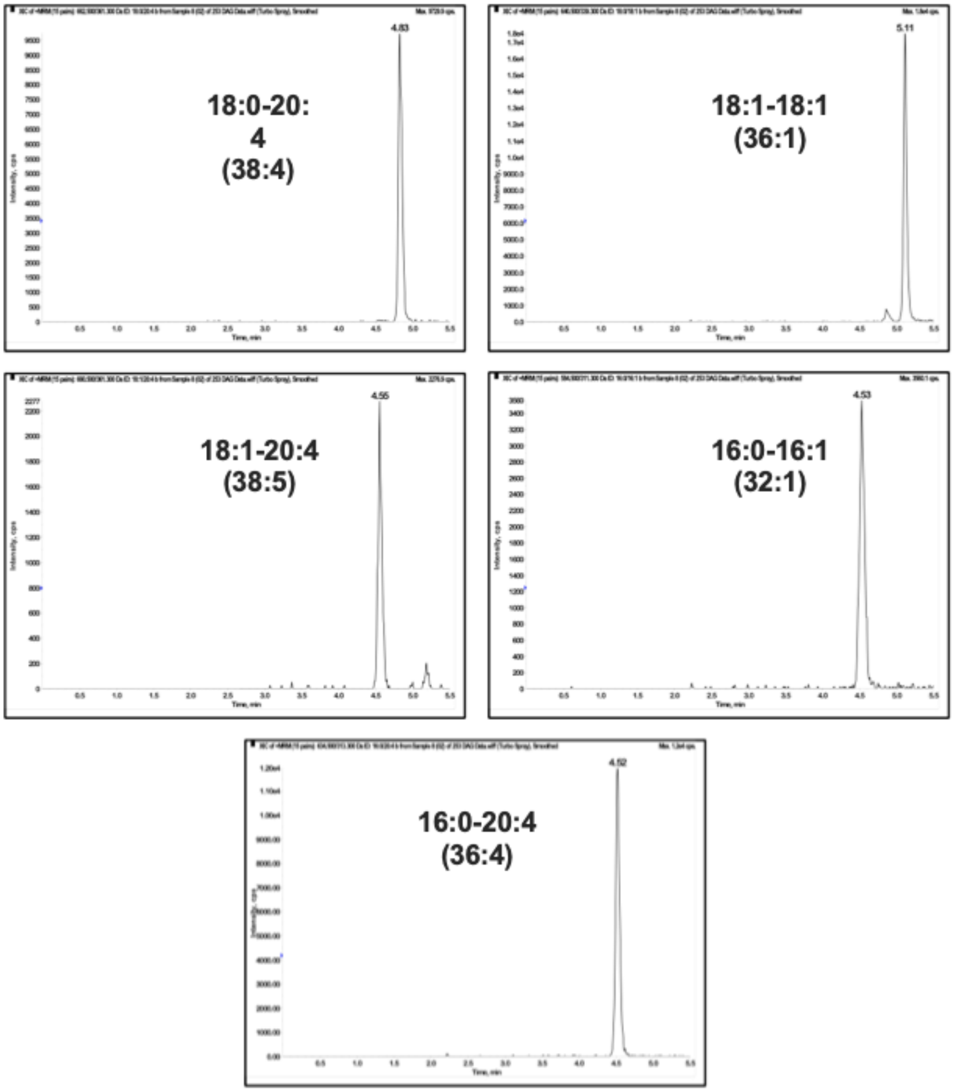

Fig. S5. LC-MS/MS chromatograms of DAG species: These representative chromatograms were obtained from analysis of a cell pellet collected from a plate of 10^6 RAW264.7 cells as described in Materials and Methods. DAG species were analyzed on the described LC-MS/MS system in positive ion mode. The specific MRM transitions are given in Table S1, and specific chromatographic parameters are given in the table below.

**Parameter**	**Value**
Mobile Phase A	1:1 methanol:H_2O with 10 mM ammonium acetate and 0.1% formic acid
Mobile Phase B	1:1 isopropanol:acetonitrile with 10 mM ammonium acetate and 0.1% formic acid
Column	Phenomenex Luna(2) C18 5 x 0.2 cm, 3 um @ 43C
Gradient	65%B to 100% B in 4.3 min

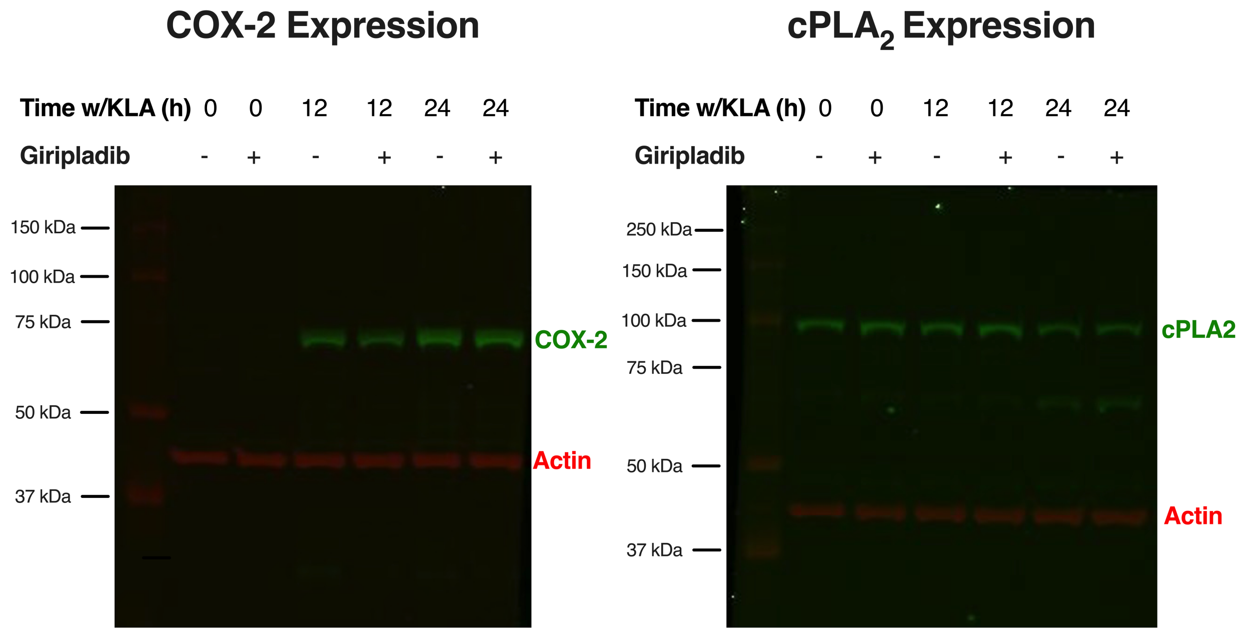

Fig. S6. COX-2 and cPLA2 protein levels following KLA treatment (12 h and 24 h) in control and giripladib-treated RAW264.7 cells as determined by western blot.

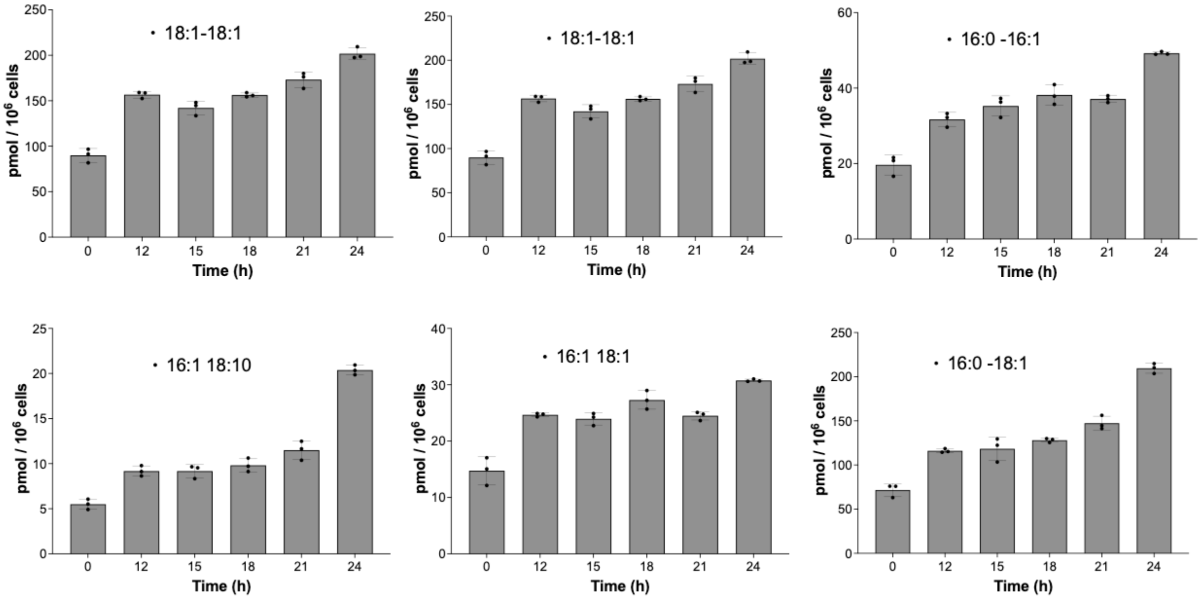

Fig. S7. RAW264.7 cells were stimulated with 100 ng/mL KLA, and the designated intracellular DAGs were analyzed by LC/MS/MS. A representative experiment is shown, and the data are expressed as mean values ± S.D.

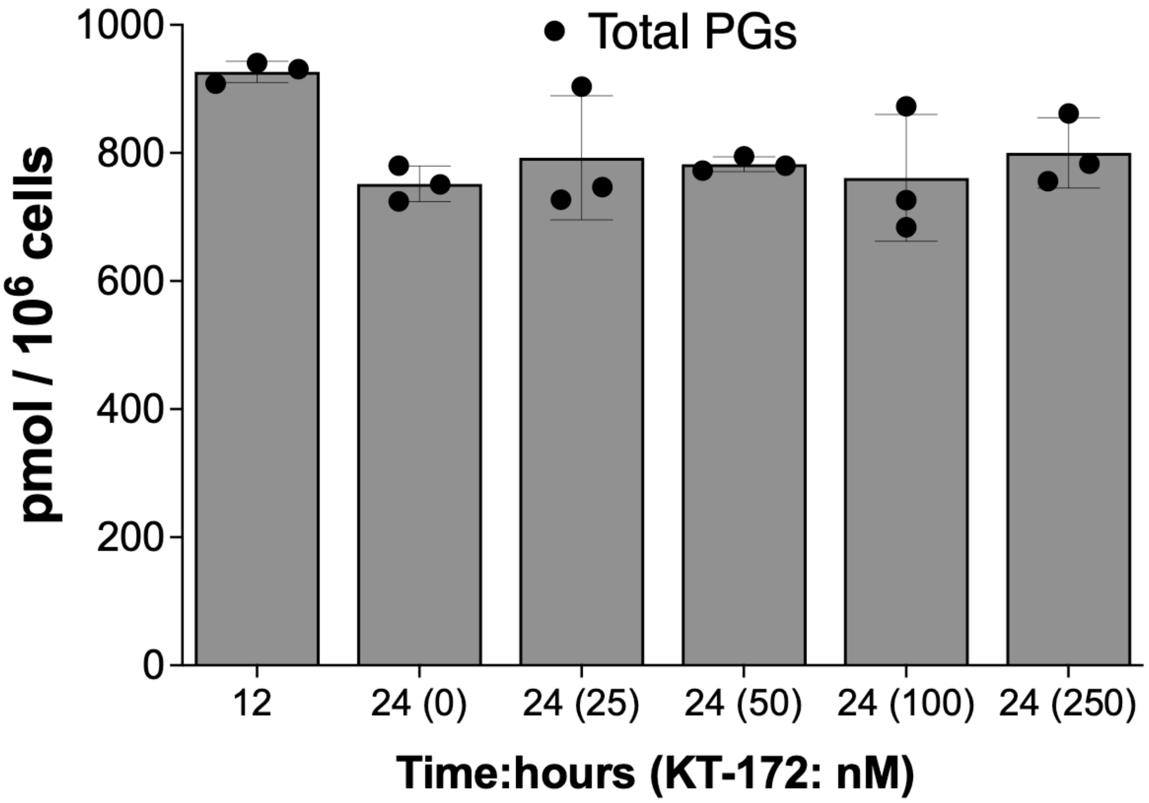

Fig. S8. Effects of inhibitor KT-172 on extracellular PGs. RAW264.7 cells were stimulated with 100 ng/mL KLA and after 12 h of activation, different concentrations (25, 50, 100 & 250 nM) of KT-172 were added to the dish. Medium was collected after 12 h of KT-172 treatment, and extracellular levels of PGs were analyzed by LC/MS/MS.

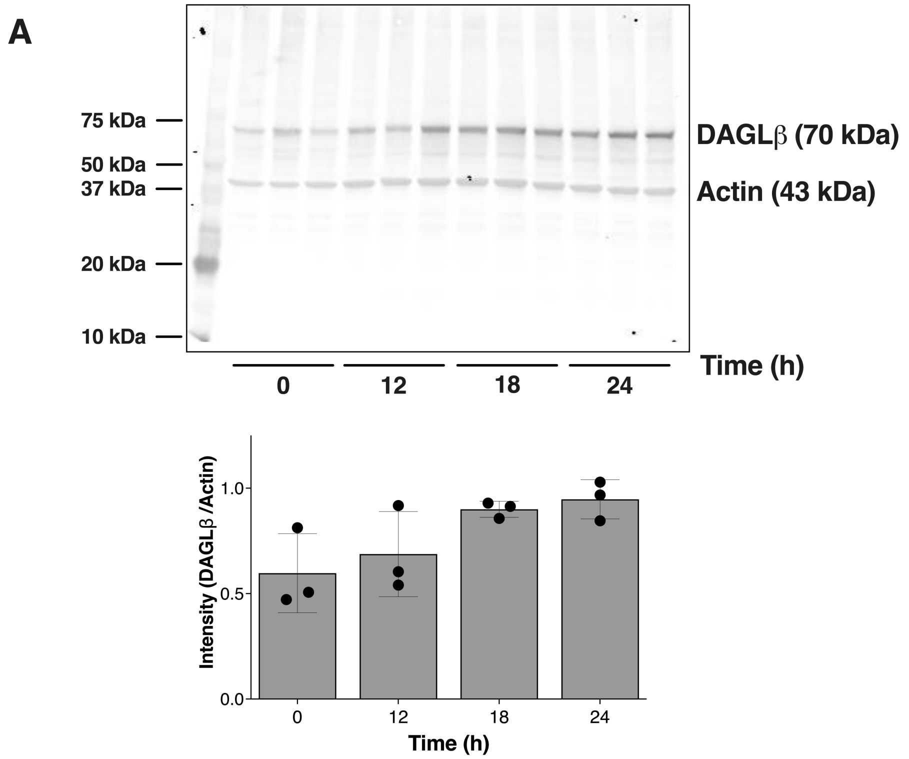

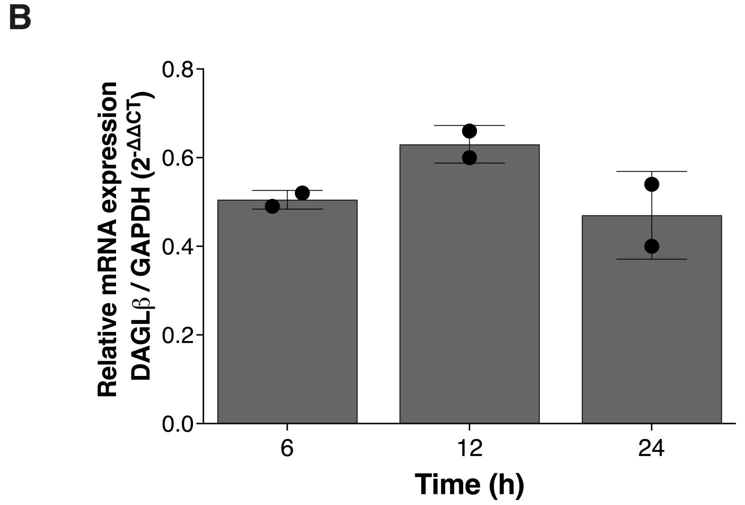

Fig. S9. DAGLβ mRNA and protein levels: A: DAGLβ protein levels at different time points (0 h, 12 h, 18 h and 24 h) were measured by western blot in cells treated with KLA along with densitometric analysis. Image J software was used to measure the intensity of the bands. B: Relative DAGLβ mRNA levels normalized to GAPDH mRNA at different time points after KLA activation of RAW264.7 cells as detected by qPCR. The values are from two different experiments with each time point performed in triplicate

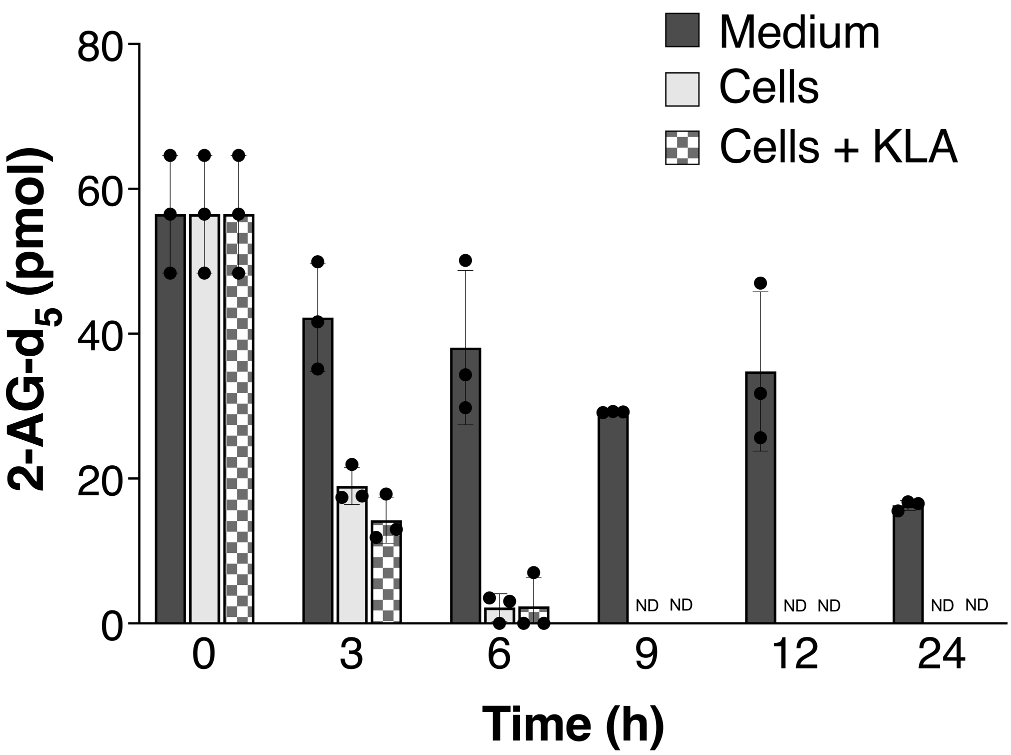

Fig. S10. Hydrolysis of 2-AG-d_5. 2-AG-d_5 (60 pmol) was added to medium alone, unactivated RAW264.7 cells, or KLA-treated RAW264.7 cells. Indomethacin (1 μM) was added to all samples to prevent oxygenation of 2-AG-d_5 by COX-2 in KLA -treated cells. The amount of 2-AG-d_5 remaining in the media at indicated time points was determined by LC-MS/MS as described in methodology section, using AA-d_8 as an internal standard. Results are the mean values ± S.D. of triplicate determinations. ND is not detected.

Table 1. MRM transitions of Analytes

Analytes	*m/z (Q1)*	*m/z (Q3)*	*Polarity*	*Q1 Ionization*
PGE_2 & D_2	351.2	271.1	-	$[M-H]^-$
$PGF_{2\alpha}$	353.2	193.1	-	$[M-H]^-$
PGJ_2 & 15d-PGD_2	333.1	271.1	-	$[M-H]^-$
15d-PGJ_2	315.2	299.1	-	$[M-H]^-$
PGE_2 & D_2-d_4	355.2	275.1	-	$[M-H]^-$
$PGF_{2\alpha}$-d_4	357.2	197.1	-	$[M-H]^-$
PGJ_2-d_4 & 15d-PGD_2-d_4	337.1	275.1	-	$[M-H]^-$
15d-PGJ_2-d_4	319.1	275.1	-	$[M-H]^-$
AA	303.3	259.1	-	$[M-H]^-$
2-AG	379.2	278.2	+	$[M+H]^+$
AA-d_8	311.2	267.1	-	$[M-H]^-$
2-AG-d_5	384.2	287.2	+	$[M+H]^+$
DAG (16:0-20:4)	634.5	313.3	+	$[M+NH_4]^+$
DAG (18:0-20:4)	662.5	341.3	+	$[M+NH_4]^+$
DAG (18:1-20:4)	660.5	339.3	+	$[M+NH_4]^+$
DAG (18:0-18:1)	640.5	341.3	+	$[M+NH_4]^+$
DAG (18:1-18:1)	638.5	339.3	+	$[M+NH_4]^+$
DAG (16:0-16:1)	584.5	313.3	+	$[M+NH_4]^+$
DAG (16:1-18:0)	612.5	341.3	+	$[M+NH_4]^+$
DAG (16:1-18:1)	610.5	339.3	+	$[M+NH_4]^+$
DAG (16:0-18:1)	612.5	313.3	+	$[M+NH_4]^+$
DAG-d_8 (18:0-20:4)	670.5	341.3	+	$[M+NH_4]^+$
PGE_2- & D_2-G	444.3	391.3	+	$[M+NH_4]^+$
PGE_2- & D_2-G-d_5	449.3	396.3	+	$[M+NH_4]^+$

References

Rouzer, C.A., K. Ghebreselasie, and L.J. Marnett, *Chemical stability of 2-arachidonylglycerol under biological conditions*. Chem Phys Lipids, 2002. **119**(1-2): p. 69-82.
